# Isoxazol-5(2*H*)-one: a new scaffold for potent human neutrophil elastase (HNE) inhibitors

**DOI:** 10.1080/14756366.2017.1326915

**Published:** 2017-06-14

**Authors:** Claudia Vergelli, Igor A. Schepetkin, Letizia Crocetti, Antonella Iacovone, Maria Paola Giovannoni, Gabriella Guerrini, Andrei I. Khlebnikov, Samuele Ciattini, Giovanna Ciciani, Mark T. Quinn

**Affiliations:** a NEUROFARBA, Sezione di Farmaceutica e Nutraceutica, Università degli Studi di Firenze, Sesto Fiorentino, Italy;; b Department of Microbiology and Immunology, Montana State University, Bozeman, MT, USA;; c Department of Biotechnology and Organic Chemistry, Tomsk Polytechnic University, Tomsk, Russia;; d Department of Chemistry, Altai State Technical University, Barnaul, Russia;; e Dipartimento di Chimica, Centro di Cristallografia, Università degli Studi di Firenze, Sesto Fiorentino, Italy

**Keywords:** Isoxazol-5(2H)-one, human neutrophil elastase, HNE inhibitor, chemical stability, molecular docking

## Abstract

Human neutrophil elastase (HNE) is an important target for the development of novel and selective inhibitors to treat inflammatory diseases, especially pulmonary pathologies. Here, we report the synthesis, structure–activity relationship analysis, and biological evaluation of a new series of HNE inhibitors with an isoxazol-5(2*H*)-one scaffold. The most potent compound (**2o**) had a good balance between HNE inhibitory activity (IC_50_ value =20 nM) and chemical stability in aqueous buffer (*t*_1/2_=8.9 h). Analysis of reaction kinetics revealed that the most potent isoxazolone derivatives were reversible competitive inhibitors of HNE. Furthermore, since compounds **2o** and **2s** contain two carbonyl groups (2-N-CO and 5-CO) as possible points of attack for Ser195, the amino acid of the active site responsible for the nucleophilic attack, docking studies allowed us to clarify the different roles played by these groups.

## Graphical Abstract

**Figure:**
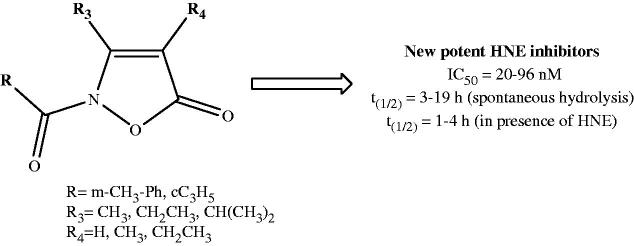

## Introduction

Human neutrophil elastase (HNE) is a member of the chymotrypsin superfamily of serine proteases and is primarily expressed in neutrophil azurophilic granules^1^^,^^2^. It is one of the few proteases able to degrade the matrix protein elastin, but can also degrade a variety of extracellular other matrix proteins, such as fibronectin, collagen, proteoglycans, and laminin^3^. Moreover, HNE plays a pivotal role both in intracellular and extracellular innate immune responses against bacteria^4^. HNE performs its proteolytic action on the membranes of Gram-negative bacteria engulfed in neutrophil phagolysosomes^5^ and in association with special structures called “neutrophil extracellular traps” (NETs) that are extruded into the extracellular space^6^. High molecular weight serine protease inhibitors called SERPINs (e.g. alpha-1 antitrypsin (AAT), elafin, and secretory leucocyte protease inhibitor) strictly regulate HNE activity to prevent uncontrolled proteolysis, and alteration of the protease–antiprotease balance^7^^,^^8^ plays an important role in some inflammatory diseases, mainly those involving the respiratory system. For example, chronic obstructive pulmonary disease (COPD)^9^ is characterised by an impaired protease–antiprotease balance. Likewise, cystic fibrosis (CF)^10^, bronchiectasis (BE), pulmonary arterial hypertension (PAH), acute lung injury (ALI), and adult respiratory distress syndrome (ARDS)^11^^,^^12^ involve imbalances in protease–antiprotease activities. HNE has also recently been implicated in the progression of various types of cancer and other important neutrophil-driven inflammatory diseases, such as rheumatoid arthritis and inflammatory bowel disease^13^.

**Figure 1.**
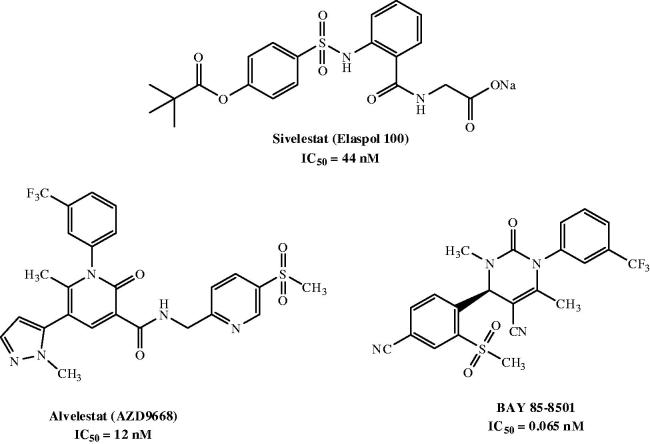
HNE inhibitors.

In the last three decades, research on the development of innovative elastase inhibitors has resulted in the synthesis of a number of new compounds, including peptide and non-peptide derivatives^14^^,^^15^. Currently, Sivelestat (Elaspol^®^ 100) is the only non-peptide drug available for the treatment of ALI/ARDS^16^, although it is only marketed in Japan and Korea. Sivelestat has been also used in paediatric heart surgery with cardiopulmonary bypass (CPB), where it attenuated the perioperative inflammatory response due to release of neutrophil proteolytic enzymes such as elastase^17^. Additionally, two potent and orally active candidates, AZD9668 (Alvelestat, Astra Zeneca, Cambridge, UK)^18^^,^^19^ and BAY 85-8501 (Bayer HealthCare, Leverkusen, Germany)^20^, are in clinical trials (Phase II) for patients with BE, CF, COPD, and pulmonary disease (Figure 1).

Our interest in the design and synthesis of new non-peptide HNE inhibitors resulted from the discovery of a potent class of HNE inhibitors with a N-benzoylindazole scaffold^21^^,^^22^. These compounds had IC_50_ values in the low nanomolar range. Studies on their mechanism of action demonstrated that these compounds were competitive and pseudo-irreversible HNE inhibitors with greater selectivity for HNE versus other serine proteases and had appreciable chemical stability in aqueous buffer. Further work led to additional interesting HNE inhibitors with cinnoline^23^ and indole^24^ scaffolds.

In the present study, we report a novel class of HNE inhibitors with an isoxazol-5(*2H*)-one core. We designed and synthesised small and flexible molecules bearing alkyl groups at positions 3 and 4 or 4-unsubstituted derivatives, also introducing those substituents identified in our previous compound series^21–23^ to be fundamental for HNE inhibitory activity.

## Materials and methods

All melting points were determined on a Büchi apparatus (New Castle, DE) and are uncorrected. Extracts were dried over Na_2_SO_4_, and the solvents were removed under reduced pressure. Merck F-254 commercial plates (Merck, Durham, NC) were used for analytical TLC to follow the course of the reactions. Silica gel 60 (Merck 70–230 mesh, Merck, Durham, NC) was used for column chromatography. ^1^H NMR, ^13^C NMR, HSQC, and HMBC spectra were recorded on an Avance 400 instrument (Bruker Biospin Version 002 with SGU, Bruker Inc., Billerica, MA). Chemical shifts (*δ*) are reported in ppm to the nearest 0.01 ppm using the solvent as an internal standard. Coupling constants (*J* values) are presented in Hz as calculated using TopSpin 1.3 software (Nicolet Instrument Corp., Madison, WI) and are rounded to the nearest 0.1 vHz. Mass spectra (*m/z*) were recorded on an ESI-TOF mass spectrometer (Brucker Micro TOF, Bruker Inc., Billerica, MA), and reported mass values are within the error limits of ±5 ppm mass units. Microanalyses indicated by the symbols of the elements or functions were performed with a Perkin–Elmer 260 elemental analyser (PerkinElmer, Inc., Waltham, MA) for C, H, and N, and the results were within ±0.4% of the theoretical values, unless otherwise stated. Reagents and starting material were commercially available. The purity of all tested compounds is >95%. The crystal structure of compound **2j** was solved by single-crystal X-ray diffraction.

*Chemistry. General procedure for synthesis of compounds (****2a–s****).* To a suspension of the appropriate substrates **1a–f**^25–29^ (0.86 mmol) in 10 ml of anhydrous THF, 1.72 mmol of sodium hydride and 1.03 mmol of the appropriate acyl/aroyl chloride were added. The mixture was stirred at room temperature overnight. The solvent was concentrated *in vacuo* to obtain the final compounds **2a–s**, which were purified by column chromatography using cyclohexane/ethyl acetate (2:1 for **2a**,**i**,**s**; 3:1 for **2e**,**g**,**h**,**j**,**k**,**p**,**q**; 4:1 for **2c**,**l**,**m**; and 5:1 for **2d**,**r**] or toluene/ethyl acetate 9.5:0.5 (**2b**,**f**,**n**,**o**) as eluents.

*2-(Cyclopropanecarbonyl)-3,4-dimethylisoxazol-5(2H)-one (****2a****).* Yield = 32%; oil. ^1^H NMR (CDCl_3_-d_1_) *δ* 1.02–1.07 (m, 2H, CH_2_ cC_3_H_5_), 1.12–1.17 (m, 2H, CH_2_ cC_3_H_5_), 1.82 (s, 3H, CH_3_), 2.34–2.40 (m, 1H, CH cC_3_H_5_), 2.49 (s, 3H, CH_3_). 13C NMR (CDCl_3_-d_1_) *δ* 6.26 (CH_3_), 10.44 (CH_2_), 12.50 (CH), 13.77 (CH_3_), 102.41 (C), 153.33 (C), 167.77 (C), 168.74 (C). ESI-MS calcd. for C_9_H_11_NO_3_, 181.19; found: *m/z* 182.08 [M + H]^+^. Anal. C_9_H_11_NO_3_ (C, H, N).

*3,4-Dimethyl-2-(3-methylbenzoyl)isoxazol-5(2H)-one (****2b****).* Yield = 30%; oil. ^1^H NMR (CDCl_3_-d_1_) *δ* 1.75 (s, 3H, CH_3_), 2.05 (s, 3H, CH_3_), 2.41 (s, 3H, *CH_3_*-Ph), 7.35 (t, 1H, Ar, *J = *8.0 Hz), 7.44 (d, 1H, Ar, *J = *7.6 Hz), 7.80–7.85 (m, 2H, Ar). 13C NMR (CDCl_3_-d_1_) *δ* 10.99 (CH_3_), 20.12 (CH_3_), 21.23 (CH_3_), 29.70 (C), 127.38 (CH), 128.65 (CH), 130.68 (CH), 135.28 (CH), 134.10 (C), 138.70 (C), 143.10 (C), 157.61 (C), 169.57 (C). ESI-MS calcd. for C_13_H_13_NO_3_, 231.25; found: *m/z* 232.09 [M + H]^+^. Anal. C_13_H_13_NO_3_ (C, H, N).

*2-(Cyclopropanecarbonyl)-4-ethyl-3-methylisoxazol-5(2H)-one (****2c****).* Yield = 26%; oil. ^1^H NMR (CDCl_3_-d_1_) *δ* 0.98–1.05 (m, 2H, CH_2_ cC_3_H_5_), 1.10 (t, 3H, CH_2_*CH_3_*, *J = *7.6 Hz), 1.13–1.18 (m, 2H, CH_2_ cC_3_H_5_), 2.26 (q, 2H, *CH_2_*CH_3_, *J = *7.6 Hz), 2.34–2.40 (m, 1H, CH cC_3_H_5_), 2.50 (s, 3H, CH_3_). 13C NMR (CDCl_3_-d_1_) *δ* 10.96 (CH_2_), 13.09 (CH_3_), 13.58 (CH_3_), 14.06 (CH), 15.53 (CH_2_), 108.58 (C), 153.56 (C), 167.87 (C), 169.34 (C). ESI-MS calcd. for C_10_H_13_NO_3_, 195.22; found: *m/z* 196.09 [M + H]^+^. Anal. C_10_H_13_NO_3_ (C, H, N).

*4-Ethyl-3-methyl-2-(3-methylbenzoyl)isoxazol-5(2H)-one (****2d****).* Yield = 34%; oil. ^1^H NMR (CDCl_3_-d_1_) *δ* 1.13 (t, 3H, CH_2_*CH_3_*, *J = *7.6 Hz), 2.31 (q, 2H, *CH_2_*CH_3_, *J = *7.6 Hz), 2.39 (s, 3H, CH_3_), 2.61 (s, 3H, *CH_3_*-Ph), 7.30–7.37 (m, 2H, Ar), 7.61–7.66 (m, 2H, Ar). ^13^C NMR (CDCl_3_-d_1_) *δ* 12.99 (CH_3_), 13.90 (CH_2_), 15.13 (CH_3_), 21.34 (CH_3_), 109.02 (C), 126.61 (CH), 127.70 (CH), 129.75 (CH), 131.41 (C), 133.78 (CH), 138.18 (C), 154.31 (C), 163.72 (C), 167.41 (C). ESI-MS calcd. for C_14_H_15_NO_3_, 245.27; found: *m/z* 246.11 [M + H]^+^. Anal. C_14_H_15_NO_3_ (C, H, N).

*2-(Cyclopropanecarbonyl)-3-ethyl-4-methylisoxazol-5(2H)-one (****2e****).* Yield = 54%; oil. ^1^H NMR (CDCl_3_-d_1_) *δ* 0.97–1.05 (m, 2H, CH_2_ cC_3_H_5_), 1.13–1.18 (m, 2H, CH_2_ cC_3_H_5_), 1.20 (t, 3H, CH_2_*CH_3_*, *J = *7.2 Hz), 1.82 (s, 3H, CH_3_), 2.34–2.40 (m, 1H, CH cC_3_H_5_), 2.90 (q, 2H, *CH_2_*CH_3_, *J = *7.2 Hz). ^13^C NMR (CDCl_3_-d_1_) *δ* 6.03 (CH), 10.37 (CH_2_), 12.06 (CH_3_), 12.66 (CH_3_), 20.74 (CH_2_), 102.22 (C), 158.53 (C), 167.89 (C), 168.23 (C). ESI-MS calcd. for C_10_H_13_NO_3_, 195.22; found: *m/z* 196.09 [M + H]^+^. Anal. C_10_H_13_NO_3_ (C, H, N).

*3-Ethyl-4-methyl-2-(3-methylbenzoyl)isoxazol-5(2H)-one (****2f****).* Yield = 10%; oil. ^1^H NMR (CDCl_3_-d_1_) *δ* 1.30 (t, 3H, CH_2_*CH_3_*, *J = *7.6 Hz), 1.85 (s, 3H, CH_3_), 2.44 (s, 3H, CH_3_-Ph), 2.64 (q, 2H, *CH_2_*CH_3_, *J = *7.6 Hz), 7.41 (t, 1H, Ar, *J = *7.6 Hz), 7.49 (d, 1H, Ar, *J = *7.6 Hz), 7.96 (d, 2H, Ar, *J = *7.6 Hz). ^13^C NMR (CDCl_3_-d_1_) *δ* 5.92 (CH_3_), 11.36 (CH_3_), 19.55 (CH_2_), 21.41 (CH_3_), 97.56 (C), 127.03 (C), 127.96 (CH), 128.76 (CH), 131.21 (CH), 135.47 (CH), 138.45 (C), 161.76 (C), 161.96 (C), 167.00 (C). ESI-MS calcd. for C_14_H_15_NO_3_, 245.27; found: *m/z* 246.11 [M + H]^+^. Anal. C_14_H_15_NO_3_ (C, H, N).

*Ethyl 2-(cyclopropanecarbonyl)-4-methyl-5-oxo-2,5-dihydroisoxazole-3-carboxylate (****2g****).* Yield = 38%; oil. ^1^H NMR (CDCl_3_-d_1_) *δ* 1.11–1.16 (m, 2H, CH_2_ cC_3_H_5_), 1.17–1.23 (m, 2H, CH_2_ cC_3_H_5_), 1.35 (t, 3H, CH_2_*CH_3_*, *J = *7.2 Hz), 1.95 (s, 3H, CH_3_), 2.25–2.31 (m, 1H, CH cC_3_H_5_), 4.41 (q, 2H, *CH_2_*CH_3_, *J = *7.2 Hz). ^13^C NMR (CDCl_3_-d_1_) *δ* 6.89 (CH), 10.91 (CH_2_), 12.40 (CH_3_), 13.88 (CH_3_), 63.22 (CH_2_), 107.85 (C), 145.28 (C), 158.92 (C), 167.45 (C), 168.67 (C). ESI-MS calcd. for C_11_H_13_NO_5_, 239.22; found: *m/z* 240.08 [M + H]^+^. Anal. C_11_H_13_NO_5_ (C, H, N).

*Ethyl 4-methyl-2-(3-methylbenzoyl)-5-oxo-2,5-dihydroisoxazole-3-carboxylate (****2h****).* Yield = 21%; oil. ^1^H NMR (CDCl_3_-d_1_) *δ* 1.42 (t, 3H, CH_2_*CH_3_*, *J = *7.2 Hz), 2.08 (s, 3H, CH_3_), 2.44 (s, 3H, *CH_3_*-Ph), 4.45 (q, 2H, *CH_2_*CH_3_, *J = *7.2 Hz), 7.42 (t, 1H, Ar, *J = *7.6 Hz), 7.51 (d, 1H, Ar, *J = *7.6 Hz), 7.97 (d, 2H, Ar, *J = *7.6 Hz). ^13^C NMR (CDCl_3_-d_1_) *δ* 6.68 (CH_3_), 14.16 (CH_3_), 21.26 (CH_3_), 62.03 (CH_2_), 101.14 (C), 126.42 (C), 128.07 (CH), 128.90 (CH), 131.34 (CH), 135.89 (CH), 138.81 (C), 156.61 (C), 160.17 (C), 161.66 (C), 163.86 (C). ESI-MS calcd. for C_15_H_15_NO_5_, 289.28; found: *m/z* 290.10 [M + H]^+^. Anal. C_15_H_15_NO_5_ (C, H, N).

*2-(Cyclopropanecarbonyl)-3-ethylisoxazol-5(2H)-one (****2i****).* Yield = 47%; mp =92–95 °C (EtOH). ^1^H NMR (CDCl_3_-d_1_) *δ* 1.05–1.10 (m, 2H, CH_2_ cC_3_H_5_), 1.14–1.19 (m, 2H, CH_2_ cC_3_H_5_), 1.25 (t, 3H, CH_2_*CH_3_*, *J = *7.4 Hz), 2.36–2.42 (m, 1H, CH cC_3_H_5_), 2.95 (q, 2H, *CH_2_*CH_3_, *J = *7.4 Hz), 5.32 (s, 1H, CH). ^13^C NMR (CDCl_3_-d_1_) *δ* 10.83 (CH_2_), 11.33 (CH_3_), 12.69 (CH), 22.69 (CH_2_), 92.92 (CH), 164.65 (C), 166.69 (C), 168.69 (C). ESI-MS calcd. for C_9_H_11_NO_3_, 181.19; found: *m/z* 182.08 [M + H]^+^. Anal. C_9_H_11_NO_3_ (C, H, N).

*3-Ethyl-2-(3-methylbenzoyl)isoxazol-5(2H)-one (****2j****).* Yield = 48%; mp =81–84 °C (EtOH). ^1^H NMR (CDCl_3_-d_1_) *δ* 1.32 (t, 3H, CH_2_*CH_3_*, *J = *7.4 Hz), 2.40 (s, 3H, *CH_3_*-Ph), 3.09 (q, 2H, *CH_2_*CH_3_, *J = *7.4 Hz), 5.41 (s, 1H, CH), 7.33–7.40 (m, 2H, Ar), 7.64–7.69 (m, 2H, Ar). ^13^C NMR (CDCl_3_-d_1_) *δ* 11.49 (CH_3_), 21.36 (CH_3_), 23.07 (CH_2_), 93.81 (CH), 127.02 (CH), 128.28 (CH), 130.23 (CH), 131.04 (C), 134.05 (CH), 138.28 (C), 163.38 (C), 166.20 (C), 166.66 (C). ESI-MS calcd. for C_13_H_13_NO_3_, 231.25; found: *m/z* 232.09 [M + H]^+^. Anal. C_13_H_13_NO_3_ (C, H, N).

*3-Isopropyl-2-propionylisoxazol-5(2H)-one (****2k****).* Yield = 47%; mp = 92–95 °C (EtOH). ^1^H NMR (CDCl_3_-d_1_) *δ* 1.15 (t, 3H, CH_2_*CH_3_*, *J = *7.4 Hz), 1.23 (d, 6H, CH*(CH_3_)_2_*, *J = *6.8 Hz), 2.72 (q, 2H, *CH_2_*CH_3_, *J = *7.4 Hz), 3.50–3.57 (m, 1H, *CH*(CH_3_)_2_), 5.27 (s, 1H, CH). ^13^C NMR (CDCl_3_-d_1_) *δ* 7.73 (CH_3_), 21.08 (CH_3_), 28.10 (CH), 28.54 (CH_2_), 91.70 (CH), 166.50 (C), 168.45 (C), 169.51 (C). ESI-MS calcd. for C_9_H_13_NO_3_, 183.20; found: *m/z* 184.09 [M + H]^+^. Anal. C_9_H_13_NO_3_ (C, H, N).

*2-(Cyclopropanecarbonyl)-3-isopropylisoxazol-5(2H)-one (****2l****).* Yield = 27%; mp = 55–57 °C (EtOH). ^1^H NMR (CDCl_3_-d_1_) *δ* 1.07–1.12 (m, 2H, CH_2_ cC_3_H_5_), 1.16–1.21 (m, 2H, CH_2_ cC_3_H_5_), 1.26 (d, 6H, CH*(CH_3_)_2_*, *J = *6.8 Hz), 2.38–2.45 (m, 1H, CH cC_3_H_5_), 3.54–3.61 (m 1H, *CH*(CH_3_)_2_), 5.33 (s, 1H, CH). ^13^C NMR (CDCl_3_-d_1_) *δ* 10.88 (CH_2_), 12.94 (CH), 21.17 (CH_3_), 28.18 (CH), 91.72 (CH), 166.50 (C), 168.45 (C), 169.51 (C). ESI-MS calcd. for C_10_H_13_NO_3_, 195.22; found: *m/z* 196.09 [M + H]^+^. Anal. C_10_H_13_NO_3_ (C, H, N).

*2-(Cyclopentanecarbonyl)-3-isopropylisoxazol-5(2H)-one (****2m****).* Yield = 23%; oil. ^1^H NMR (CDCl_3_-d_1_) *δ* 1.26 (d, 6H, CH*(CH_3_)_2_*, *J = *6.8 Hz), 1.63–1.74 (m, 4H, 2 × CH_2_ cC_5_H_9_), 1.82–1.87 (m, 2H, CH_2_ cC_5_H_9_), 1.97–2.02 (m, 2H, CH_2_ cC_5_H_9_), 3.32–3.37 (m, 1H, CH cC_5_H_9_), 3.57–3.62 (m, 1H, *CH*(CH_3_)_2_), 5.30 (s, 1H, CH). ^13^C NMR (CDCl_3_-d_1_) *δ* 21.12 (CH_3_), 25.95 (CH_2_), 28.17 (CH), 29.56 (CH_2_), 43.61 (CH), 91.66 (CH), 166.68 (C), 169.74 (C), 170.93 (C). ESI-MS calcd. for C_12_H_17_NO_3_, 223.27; found: *m/z* 224.12 [M + H]^+^. Anal. C_12_H_17_NO_3_ (C, H, N).

*3-Isopropyl-2-(3-methylbenzoyl)isoxazol-5(2H)-one (****2n****).* Yield = 17%; oil. ^1^H NMR (CDCl_3_-d_1_) *δ* 1.34 (d, 6H, CH*(CH_3_)_2_*, *J = *6.8 Hz), 2.41 (s, 3H, *CH_3_*-Ph), 3.70–3.78 (m 1H, *CH*(CH_3_)_2_), 5.42 (s, 1H, CH), 7.33–7.41 (m, 2H, Ar), 7.62–7.67 (m, 2H, Ar). ^13^C NMR (CDCl_3_-d_1_) *δ* 21.44 (CH_3_), 28.50 (CH), 92.85 (CH), 127.15 (CH), 128.30 (CH), 130.23 (CH), 131.38 (C), 134.08 (CH), 138.32 (C), 163.56 (C), 166.83 (C), 171.07 (C). ESI-MS calcd. for C_14_H_15_NO_3_, 245.27; found: *m/z* 246.11 [M + H]^+^. Anal. C_14_H_15_NO_3_ (C, H, N).

*3-Isopropyl-2-(4-methylbenzoyl)isoxazol-5(2H)-one (****2o****).* Yield = 45%; mp =77–79 °C (EtOH). ^1^H NMR (CDCl_3_-d_1_) *δ* 1.33 (d, 6H, CH*(CH_3_)_2_*, *J = *7.0 Hz), 2.41 (s, 3H, *CH_3_*-Ph), 3.66–3.79 (m 1H, *CH*(CH_3_)_2_), 5.40 (s, 1H, CH), 7.26 (d, 2H, Ar, *J = *8.0 Hz), 7.78 (d, 2H, Ar, *J = *8.0 Hz). ^13^C NMR (CDCl_3_-d_1_) *δ* 21.91 (CH_3_), 22.30 (CH_3_), 29.05 (CH), 93.10 (CH), 129.04 (C), 129.66 (CH), 130.69 (CH), 144.82 (C), 163.72 (C), 167.36 (C), 171.67 (C). ESI-MS calcd. for C_14_H_15_NO_3_, 245.27; found: *m/z* 246.11 [M + H]^+^. Anal. C_14_H_15_NO_3_ (C, H, N).

*3-(3-Isopropyl-5-oxo-2,5-dihydroisoxazole-2-carbonyl)benzonitrile (****2p****).* Yield = 14%; oil. ^1^H NMR (CDCl_3_-d_1_) *δ* 1.31 (d, 6H, CH*(CH_3_)_2_*, *J = *6.8 Hz), 3.00–3.09 (m, 1H, *CH*(CH_3_)_2_), 6.09 (s, 1H, CH), 7.70 (t, 1H, Ar, *J = *8.0 Hz), 7.96 (d, 1H, Ar, *J = *8.0 Hz), 8.41 (d, 1H, Ar, *J = *8.0 Hz), 8.46 (s, 1H, Ar). ^13^C NMR (CDCl_3_-d_1_) *δ* 21.92 (CH_3_), 28.09 (CH), 86.78 (CH), 114.41 (C), 117.84 (C), 129.29 (C), 130.69 (CH), 134.65 (CH), 134.98 (CH), 138. 19 (CH), 159.16 (C), 164.82 (C), 172.17 (C). IR = 1600 cm^−1^ (C=O amide), 1777 cm^−1^ (C=O ester), 2235 cm^−1^ (CN). ESI-MS calcd. for C_14_H_12_N_2_O_3_, 256.26; found: *m/z* 257.09 [M + H]^+^. Anal. C_14_H_12_N_2_O_3_ (C, H, N).

*4-(3-Isopropyl-5-oxo-2,5-dihydroisoxazole-2-carbonyl)benzonitrile (****2q***). Yield = 38%; mp = 113–115 °C (EtOH). ^1^H NMR (CDCl_3_-d_1_) *δ* 1.30 (d, 6H, CH*(CH_3_)_2_*, *J = *7.2 Hz), 3.00–3.09 (m 1H, *CH*(CH_3_)_2_), 6.10 (s, 1H, CH), 7.84 (d, 2H, Ar, *J = *8.4 Hz), 8.29 (d, 2H, Ar, *J = *8.0 Hz). ^13^C NMR (CDCl_3_-d_1_) *δ* 21.29 (CH_3_), 27.48 (CH), 86.16 (CH), 117.46 (C), 118.11 (C), 131.03 (CH), 132.72 (CH), 158.88 (C), 164.28 (C), 171.60 (C). ESI-MS calcd. for C_14_H_12_N_2_O_3_, 256.26; found: *m/z* 257.09 [M + H]^+^. Anal. C_14_H_12_N_2_O_3_ (C, H, N).

*3-Isopropyl-2-(3-(trifluoromethyl)benzoyl)isoxazol-5(2H)-one (****2r****).* Yield = 21%; oil. ^1^H NMR (CDCl_3_-d_1_) *δ* 1.39 (d, 6H, CH*(CH_3_)_2_*, *J = *6.8 Hz), 3.73–3.78 (m, 1H, *CH*(CH_3_)_2_), 5.51 (s, 1H, CH), 7.66 (t, 1H, Ar, *J = *7.6 Hz), 7.87 (d, 1H, Ar, *J = *8.0 Hz), 8.09 (d, 1H, Ar, *J = *8.0 Hz), 8.14 (s, 1H, Ar). ^13^C NMR (CDCl_3_-d_1_) *δ* 21.83 (CH_3_), 29.12 (CH), 94.07 (CH), 127.33 (C), 129.64 (CH), 130.20 (C), 131.93 (C), 132.87 (CH), 133.45 (CH), 162.29 (C), 166.72 (C), 171.54 (C). ESI-MS calcd. for C_14_H_12_F_3_NO_3_, 299.25; found: *m/z* 300.08 [M + H]^+^. Anal. C_14_H_12_F_3_NO_3_ (C, H, N).

*3-Isopropyl-2-(4-(trifluoromethyl)benzoyl)isoxazol-5(2H)-one (****2s****).* Yield = 16%; oil. ^1^H NMR (CDCl_3_-d_1_) *δ* 1.36 (d, 6H, CH*(CH_3_)_2_*, *J = *6.8 Hz), 3.69–3.77 (m 1H, *CH*(CH_3_)_2_), 5.47 (s, 1H, CH), 7.75 (d, 2H, Ar, *J = *8.0 Hz), 7.97 (d, 2H, Ar, *J = *8.0 Hz). ^13^C NMR (CDCl_3_-d_1_) *δ* 21.24 (CH_3_), 28.51 (CH), 93.46 (CH), 125.40 (CH), 127.30 (C), 130.09 (CH), 130.20 (C), 134.72 (C), 161.81 (C), 166.10 (C), 170.86 (C). ESI-MS calcd. for C_14_H_12_F_3_NO_3_, 299.25; found: *m/z* 300.08 [M + H]^+^. Anal. C_14_H_12_F_3_NO_3_ (C, H, N).

*General procedure for synthesis of compounds (****3a,b****).* To a suspension of the appropriate substrate **2l** or **2n** (0.36 mmol) in 5 ml of anhydrous toluene, 0.72 mmol of Lawesson’s reagent were added. The mixture was stirred at reflux for 6 h, cooled, concentrated *in vacuo*, diluted with ice-cold water (10 ml), and extracted with ethyl acetate (3 × 15 ml). The organic phase was dried over sodium sulphate, and the solvent was evaporated *in vacuo* to obtain the final compounds **3a,b**, which were purified by column chromatography using cyclohexane/ethyl acetate as eluent (ratio: 5:1 for **3a** and 3:1 for **3b**).

*Cyclopropyl-(3-isopropyl-5-thioxoisoxazol-2(5H)-yl)methanone (****3a****).* Yield = 26%; oil. ^1^H NMR (CDCl_3_-d_1_) *δ* 1.17–1.24 (m, 4H, 2 × CH_2_ cC_3_H_5_), 1.27 (d, 6H, CH*(CH_3_)_2_*, *J = *6.8 Hz), 2.55–2.61 (m, 1H, CH cC_3_H_5_), 3.54–3.61 (m, 1H, *CH*(CH_3_)_2_), 6.12 (s, 1H, CH). ^13^C NMR (CDCl_3_-d_1_) *δ* 10.88 (CH_2_), 14.76 (CH), 21.15 (CH_3_), 35.40 (CH), 106.37 (CH), 166.84 (C), 169.52 (C), 171.69 (C). ESI-MS calcd. for C_10_H_13_NO_2_S, 211.28; found: *m/z* 212.07 [M + H]^+^. Anal. C_10_H_13_NO_2_S (C, H, N).

*(3-Isopropyl-5-thioxoisoxazol-2(5H)-yl)-(m-tolyl)methanone (****3b****).* Yield = 40%; oil. ^1^H NMR (CDCl_3_-d_1_) *δ* 1.32 (d, 6H, CH*(CH_3_)_2_*, *J = *6.4 Hz), 2.44 (s, 3H, *CH_3_*-Ph), 2.83–2.92 (m, 1H, *CH*(CH_3_)_2_), 7.05 (s, 1H, CH), 7.36–7.41 (m, 2H, Ar), 7.81–7.86 (m, 2H, Ar). ^13^C NMR (CDCl_3_-d_1_) *δ* 21.09 (CH_3_), 37.59 (CH_3_), 108.44 (CH), 122.51 (CH), 124.40 (CH), 127.37 (CH), 129.54 (CH), 131.38 (C), 134.14 (CH), 138.32 (C), 163.56 (C), 169.76 (C), 175.11 (C). ESI-MS calcd. for C_14_H_15_NO_2_S, 261.34; found: *m/z* 262.09 [M + H]^+^. Anal. C_14_H_15_NO_2_S (C, H, N).

*General procedure for synthesis of compounds (****4a,b****).* A mixture of the appropriate intermediates (**1a**^25^ or **1f**^29^) (0.63 mmol), K_2_CO_3_ (1.26 mmol), and 1-(chloromethyl)-3-methylbenzene (0.95 mmol) in 2 ml of anhydrous acetonitrile was stirred at reflux for 2 h. After cooling, the mixture was concentrated *in vacuo*, diluted with ice-cold water (10 ml), and extracted with ethyl acetate (3 × 15 ml). The organic phase was dried over sodium sulphate, and the solvent was evaporated *in vacuo* to obtain the final compounds **4a,b**, which were purified by column chromatography using cyclohexane/ethyl acetate as eluent (ratio: 1:1 for **4a** and 2:1 for **4b**).

*3,4-Dimethyl-2-(3-methylbenzyl)isoxazol-5(2H)-one (****4a****).* Yield = 26%; mp = 80–83 °C (EtOH). ^1^H NMR (CDCl_3_-d_1_) *δ* 1.73 (s, 3H, CH_3_), 2.13 (s, 3H, CH_3_), 2.32 (s, 3H, *CH_3_*-Ph), 4.59 (s, 2H, CH_2_), 7.03–7.12 (m, 3H, Ar), 7.20 (t, 1H, Ar, *J = *7.6 Hz). ^13^C NMR (CDCl_3_-d_1_) *δ* 6.75 (CH_3_), 11.30 (CH_3_), 29.70 (CH_3_), 55.18 (CH_2_), 100.16 (C), 125.42 (CH), 128.63 (CH), 129.10 (CH), 129.19 (CH), 133.40 (C), 138.50 (C), 160.32 (C), 171.85 (C). ESI-MS calcd. for C_13_H_15_NO_2_, 217.26; found: *m/z* 218.11 [M + H]^+^. Anal. C_13_H_15_NO_2_ (C, H, N).

*3-Isopropyl-2-(3-methylbenzyl)isoxazol-5(2H)-one (****4b****).* Yield = 14%; oil. ^1^H NMR (CDCl_3_-d_1_) *δ* 1.24 (d, 6H, CH*(CH_3_)_2_*, *J = *6.8 Hz), 2.33 (s, 3H, *CH_3_*-Ph), 2.73–2.79 (m, 1H, *CH*(CH_3_)_2_), 4.74 (s, 2H, CH_2_), 5.00 (s, 1H, CH), 7.02–7.07 (m, 2H, Ar), 7.12 (d, 1H, Ar, *J = *7.6 Hz), 7.22 (t, 1H, Ar, *J = *7.4 Hz). ^13^C NMR (CDCl_3_-d_1_) *δ* 21.41 (CH_3_), 21.60 (CH_3_), 26.58 (CH), 56.00 (CH_2_), 81.50 (CH), 124.93 (CH), 129.40 (CH), 133.87 (CH), 138.22 (C), 138.81 (CH), 141.50 (C), 171.51 (C), 174.09 (C). ESI-MS calcd. for C_14_H_17_NO_2_, 231.29; found: *m/z* 232.13 [M + H]^+^. Anal. C_14_H_17_NO_2_ (C, H, N).

*Procedure for synthesis of ethyl 4-methyl-5-oxo-2-(m-tolyl)-2,5-dihydroisoxazole-3-carboxylate (****4c****).* A mixture of dry CH_2_Cl_2_ (8 ml), **1d**^28^ (0.58 mmol), 3-methylphenylboronic acid (1.16 mmol), Cu(Ac)_2_ (0.87 mmol), and Et_3_N (0.87 mmol) was stirred at room temperature for 24 h. The organic layer was washed with water (3 × 20 ml), then with 33% aqueous ammonia (3 × 5 ml). The organic phase was dried over sodium sulphate, and the solvent was evaporated *in vacuo* to obtain the final compound **4c**, which was purified by column chromatography using cyclohexane/ethyl acetate 3:1 as eluent. Yield = 59%; oil. ^1^H NMR (CDCl_3_-d_1_) *δ* 1.16 (t, 3H, CH_2_*CH_3_*, *J = *7.2 Hz), 2.16 (s, 3H, CH_3_), 2.36 (s, 3H, *CH_3_*-Ph), 4.23 (q, 2H, *CH_2_*CH_3,_*J = *7.2 Hz), 7.03–7.08 (m, 2H, Ar), 7.20 (d, 1H, Ar, *J = *7.6 Hz), 7.28 (t, 1H, Ar, *J = *7.6 Hz). ^13^C NMR (CDCl_3_-d_1_) *δ* 8.38 (CH_3_), 14.06 (CH_3_), 21.36 (CH_3_), 62.47 (CH_2_), 111.35 (C), 122.73 (CH), 126.24 (CH), 129.03 (CH), 130.60 (CH), 139.48 (C), 140.23 (C), 150.44 (C), 158.63 (C), 171.24 (C). ESI-MS calcd. for C_14_H_15_NO_4_, 261.27; found: *m/z* 262.10 [M + H]^+^. Anal. C_14_H_15_NO_4_ (C, H, N).

*HNE inhibition assay.* Compounds were dissolved in 100% DMSO at 5 mM stock concentrations. The final concentration of DMSO in the reactions was 1%, and this level of DMSO had no effect on enzyme activity. The HNE inhibition assay was performed in black flat-bottom 96-well microtiter plates. Briefly, a buffer solution containing 200 mM Tris–HCl, pH 7.5, 0.01% bovine serum albumin, 0.05% Tween-20, and 20 mU/ml of HNE (Calbiochem, La Jolla, CA) was added to wells containing different concentrations of each compound. The reaction was initiated by addition of 25 μM elastase substrate (N-methylsuccinyl-Ala-Ala-Pro-Val-7-amino-4-methylcoumarin, Calbiochem) in a final reaction volume of 100 μL/well. Kinetic measurements were obtained every 30 s for 10 min at 25 °C using a Fluoroskan Ascent FL fluorescence microplate reader (Thermo Electron, Waltham, MA) with excitation and emission wavelengths at 355 and 460 nm, respectively. For all compounds tested, the concentration of inhibitor that caused 50% inhibition of the enzymatic reaction (IC_50_) was calculated by plotting % inhibition versus logarithm of inhibitor concentration (at least six points). The data are presented as the mean values of at least three independent experiments with relative standard deviations of <15%.

### Analysis for compound stability

Spontaneous hydrolysis of selected derivatives was evaluated at 25 °C in 0.05 M phosphate buffer, pH 7.3. Kinetics of hydrolysis were monitored by measuring changes in absorbance spectra over time using a SpectraMax Plus microplate spectrophotometer (Molecular Devices, Sunnyvale, CA). Absorbance (*A*_t_) at the characteristic absorption maxima of each compound was measured at the indicated times until no further absorbance decreases occurred (*A*_∞_)^30^. Using these measurements, we created semilogarithmic plots of log(*A*_t_ – *A*_∞_) versus time, and *k*′ values were determined from the slopes of these plots. Half-conversion times were calculated using *t*_1/2_=0.693/*k*′, as described previously^21^.

### Molecular modelling

Initial structures of the compounds were generated with HyperChem 8.0 (Shimadzu Corporation, Kyoto, Japan) and optimised by the semi-empirical PM3 method. Docking of the molecules was performed using Molegro Virtual Docker (CLC Bio, København, Denmark) (MVD), version 4.2.0 (CLC Bio, København, Denmark), as described previously^22^. The structure of HNE complexed with a peptide chloromethyl ketone inhibitor^31^ was used for the docking study (1HNE entry of the Protein Data Bank). The search area for docking poses was defined as a sphere with a 10 Å radius centred at the nitrogen atom in the five-membered ring of the peptide chloromethyl ketone inhibitor. After removal of this peptide and co-crystallised water molecules from the program workspace, we set side chain flexibility for the 42 residues closest to the centre of the search area^22^. Fifteen docking runs were performed for each compound, with full flexibility of a ligand around all rotatable bonds and side chain flexibility of the above-mentioned residues of the enzyme. Parameters used within Docking Wizard of the Molegro program were as described previously^22^. The docking poses corresponding to the lowest-energy binding mode of each inhibitor were evaluated for the ability to form a Michaelis complex between the hydroxyl group of Ser195 and the carbonyl group in the amido moiety of an inhibitor. For this purpose, values of *d*_1_ [distance O(Ser195)·C between the Ser195 hydroxyl oxygen atom and the inhibitor carbonyl carbon atom closest to O(Ser195)] and *α* [angle O(Ser195)···C=O, where C=O is the carbonyl group of an inhibitor closest to O(Ser195)] were determined for each docked compound^32^. In addition, we estimated the possibility of proton transfer from Ser195 to Asp102 through His57 (the key catalytic triad of serine proteases) by calculating distances *d*_2_ between the NH hydrogen in His57 and carboxyl oxygen atoms in Asp102, as described previously^22^. The distance between the hydroxyl proton in Ser195 and the pyridine-type nitrogen in His57 is also important for proton transfer. However, because of easy rotation of the hydroxyl about the C–O bond in Ser195, we measured distance *d*_3_ between the oxygen in Ser195 and the basic nitrogen atom in His57. The effective length *L* of the channel for proton transfer was calculated as *L*=*d*_3_+min (*d*_2_).

### Crystallographic analysis

The data were collected at 100(2) K on Xcalibur3 CCD 4-circle diffractometer using a graphite monochromator, Mo Ka radiation. A reference frame was monitored every 50 frames to control the stability of the crystal, and the system revealed no intensity decay. The data set was corrected for Lorentz, polarisation effects, and absorption corrections were performed by the ABSPACK^33^ multi-scan procedure of the CrysAlis data reduction package. The structure was solved using the direct method with SUPERFLIP^34^ software, and the refinement was carried out using the SHELXL-2013^35^ software package. All non-hydrogen atoms were located from the initial solution or from subsequent electron density difference maps during the initial course of the refinement. After locating the non-hydrogen atoms, the models were refined against *F*^2^, first using isotropic and finally anisotropic thermal displacement parameters. Hydrogen atoms have been introduced as “riding hydrogens”. Programs used in the crystallographic calculations included WinGX4^36^ and Mercury^37^ for graphics. Crystal structural data are available from the Cambridge Crystallographic CCDC 1036557.

## Results and discussion

### Chemistry

**Figure 2.**
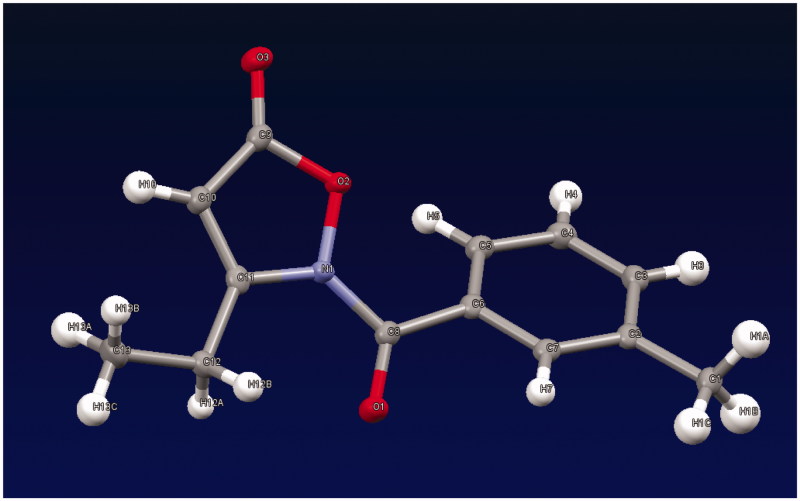
X-ray structure of compound **2j**.

**Scheme 1.**
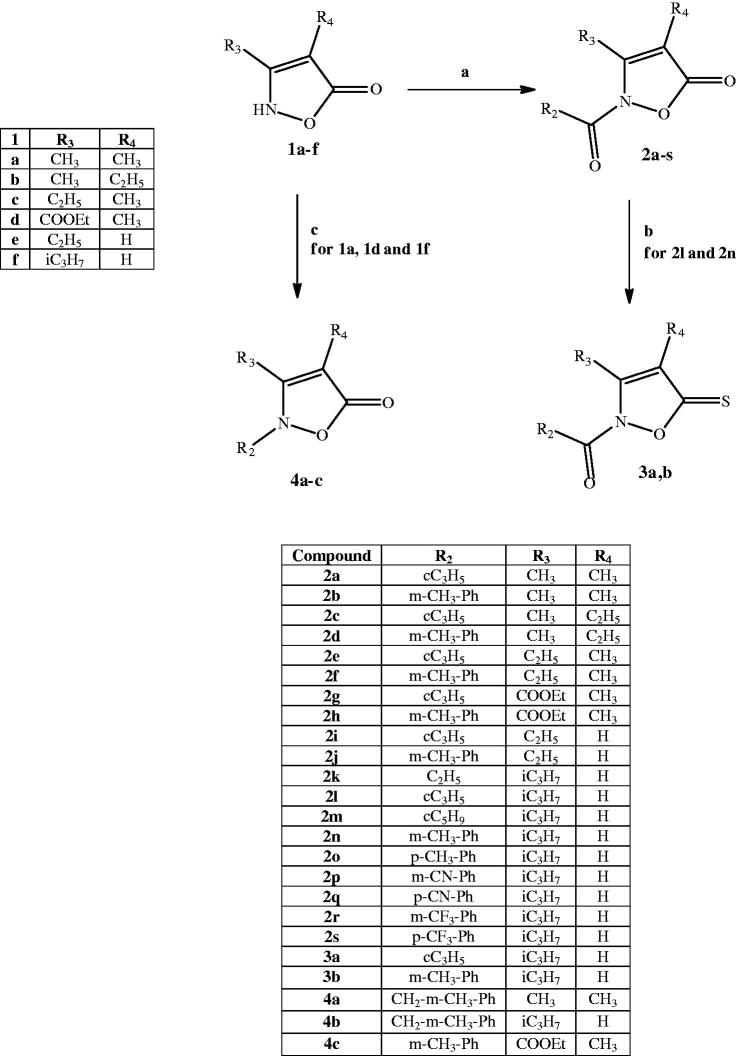
Reagents and conditions: (a) R_2_-COCl, NaH, anhydrous THF, r.t., 24 h; (b) Lawesson’s reagent, toluene, reflux, 5 h; (c) for 4a,b: 3-methylbenzyl chloride, K_2_CO_3_, anhydrous CH_3_CN, 80 °C, 2 h; for 4c: 3-methylphenyl boronic acid, (CH_3_COO)_2_Cu, Et_3_N, anhydrous CH_2_Cl_2_, r.t., 24 h.

The synthetic pathways leading to compounds **2a–s**, **3a,b**, and **4a–c** are shown in Scheme 1. Compounds of type **2** were obtained by treatment of the isoxazolones **1a–f**^25–29^ with the appropriate acyl/aroyl chloride and sodium hydride in anhydrous tetrahydrofuran. Compounds **2l** and **2n** were further converted into the corresponding thioxoisoxazolones **3a,b** with Lawesson’s reagent. Alkylation of **1a** and **1f** with 3-methylbenzyl chloride in anhydrous acetonitrile and K_2_CO_3_ and of **1d** with 3-methylphenyl boronic acid, copper acetate, and triethylamine in anhydrous dichloromethane by a coupling reaction resulted in **4a**,**b** and **4c**, respectively. All of the final compounds were identified by ^1^H NMR and ^13^C NMR. Moreover, heteronuclear multiple bond correlation (HMBC) and heteronuclear single quantum coherence (HSQC) two-dimensional NMR analyses were performed for representative products. Finally, since the literature describes the presence of tautomer's of the isozazol-*5(2H)-*one nucleus^38–40^, a crystallographic analysis of **2j** (Figure 2) was performed in order to univocally assign the structure (see Supplemental data).

### Biological evaluation and structure–activity relationship (SAR) analysis

All compounds were evaluated for their ability to inhibit HNE in comparison with Sivelestat, a reference HNE inhibitor, and the results presented in Table 1 demonstrated that the isoxazolone nucleus is an appropriate scaffold for HNE inhibitor development. These compounds display two carbonyl groups (2-N-CO and 5-CO) as possible points of attack for Ser195, the amino acid of the HNE active site responsible for the nucleophilic attack. The observation that all 2-N-acyl/aroyl derivatives were active HNE inhibitors (**2a–s**), while the 2-N-alkyl/aryl derivatives were completely inactive (**4a–c**), seems to suggest that the carbonyl group involved in the catalysis is the amidic carbonyl at position *2*. Indeed, this observation is consistent with our previous results for indazole derivatives^21^^,^^22^.

**Table 1.** HNE inhibitory activity of isoxazolone derivatives **2a–s**, **3a,b**, **4a–c**.


Compound	R_2_	R_3_	R_4_	IC_50_ (μM)^a^
**2a**	cC_3_H_5_	CH_3_	CH_3_	0.12 ± 0.023
**2b**	m-CH_3_-Ph	CH_3_	CH_3_	10.9 ± 1.2
**2c**	cC_3_H_5_	CH_3_	C_2_H_5_	0.13 ± 0.025
**2d**	m-CH_3_-Ph	CH_3_	C_2_H_5_	0.094 ± 0.023
**2e**	cC_3_H_5_	C_2_H_5_	CH_3_	2.2 ± 0.28
**2f**	m-CH_3_-Ph	C_2_H_5_	CH_3_	1.8 ± 0.32
**2g**	cC_3_H_5_	COOEt	CH_3_	0.44 ± 0.14
**2h**	m-CH_3_-Ph	COOEt	CH_3_	39.7 ± 5.1
**2i**	cC_3_H_5_	C_2_H_5_	H	0.096 ± 0.022
**2j**	m-CH_3_-Ph	C_2_H_5_	H	0.043 ± 0.011
**2k**	C_2_H_5_	iC_3_H_7_	H	1.1 ± 0.14
**2l**	cC_3_H_5_	iC_3_H_7_	H	0.034 ± 0.009
**2m**	cC_5_H_9_	iC_3_H_7_	H	0.56 ± 0.15
**2n**	m-CH_3_-Ph	iC_3_H_7_	H	0.042 ± 0.011
**2o**	p-CH_3_-Ph	iC_3_H_7_	H	0.020 ± 0.007
**2p**	m-CN-Ph	iC_3_H_7_	H	6.5 ± 1.7
**2q**	p-CN-Ph	iC_3_H_7_	H	18.2 ± 2.5
**2r**	m-CF_3_-Ph	iC_3_H_7_	H	1.2 ± 0.22
**2s**	p-CF_3_-Ph	iC_3_H_7_	H	0.034 ± 0.012
**3a**	cC_3_H_5_	–	–	2.1 ± 0.31
**3b**	m-CH_3_-Ph	–	–	NA^b^
**4a**	CH_2_-m-CH_3_-Ph	CH_3_	CH_3_	NA^b^
**4b**	CH_2_-m-CH_3_-Ph	iC_3_H_7_	H	NA^b^
**4c**	m-CH_3_-Ph	COOEt	CH_3_	NA^b^
**Sivelestat**	–	–	–	0.050 ± 0.020

a IC_50_ values are presented as the mean ± SD of three independent experiments.

b NA: no inhibitory activity was found at the highest concentration of compound tested (50 μM).

The presence of small alkyl groups at positions 3 and 4 (**2a–f**) led to compounds with HNE inhibitory activity in the micromolar/submicromolar range, whereas the introduction at position 3 of a carbethoxy group (**2g,h**) gave different results, depending on the substituent at N-2. The elimination of methyl group at position 4 (**2i–2s**) resulted in compounds endowed with excellent inhibitory activity, indicating the importance of the unsubstitution of this position. This fact can be highlighted by compounds **2i** and **2j** which show 22- and 42-fold higher inhibitory activity (IC_50_=96 and 43 nM, respectively) with respect to the corresponding 4-methyl derivatives **2e** and **2f** (IC_50_=2.2 and 1.8 μM, respectively). On the other hand, in the 4-unsubstituted series, compounds **2l**, **2n**, **2o**, and **2s** also exhibited IC_50_ values in the nanomolar range (20–42 nM). Moreover, substitution of the methyl at 2-benzoyl group in the potent compounds **2n** and **2o** with a trifluoromethyl group (**2r** and **2s**) did not affect inhibitory activity when the substituent was in the *para* position of the benzoyl group (i.e. compounds **2o** and **2s** with IC_50_=20 and 34 nM, respectively) but resulted in loss of activity when the substituent was in the *meta* position.

To evaluate the importance of the 5-CO group, which does not seem to be involved in catalysis, we synthesised **3a** and **3b** as thio analogues of the two potent compounds **2l** and **2n**. However, the low activity (**3a**, IC_50_=2.1 μM) or inactivity (**3b**) of these new products clearly suggests that the 5-CO group is important for interaction with the target. To assess if this inactivity was the result of increased bulkiness and/or a weaker H-binding acceptor effect of sulphur versus oxygen in the carbonyl or if it was related to other reasons, we performed molecular modelling studies.

### Molecular modelling

To further investigate the interaction of these new compounds with HNE, molecular docking of isoxazolones derivatives **2l**, **2n**, **2o**, **2q**, **3a**, and **3b** into the HNE binding site (1HNE entry of Protein Data Bank) was performed. Side chains of selected residues in the HNE binding site were considered flexible, as described in our previous modelling studies^22^^,^^24^. According to the docking scores Δ*E* of the poses (Table 2), isoxazolones are effectively anchored within HNE binding site. However, it is well-known that catalytic activity of serine proteases is related to synchronous proton transfer from serine via histidine to aspartate residues^41^. In HNE, this catalytic triad corresponds to Ser195, His57, and Asp102, respectively. The length of the proton transfer channel is characterised by the magnitude of *L* (see Table 2). Additionally, effectiveness of HNE binding to a ligand depends on its ability to form a Michaelis complex between a carbonyl group of the ligand and an oxyanion hole centred at the hydroxyl oxygen atom of Ser195. Geometry which favours Michaelis complex formation corresponds to ligand orientation with angle *α* values from 80° to 120° and with shortened distance d_1_ (1.8–2.6 Å)^41^ (Table 2). Although a low-energy docking pose of each ligand is not completely identical to the Michaelis complex, they must have certain similarity. Hence, it is reasonable to use the geometry of the low-energy pose to evaluate possibility of complex formation.

**Table 2.** Biological activities, geometric parameters, and docking scores of the enzyme–inhibitor complexes predicted by molecular docking with MVD.

Comp.	IC_50_ (μM)^a^	α	d_1_	d_2_	d_3_	*L*^b^	Docking score Δ*E* (kcal/mol)
**2l**^c^	0.034	85.2	2.903	2.763, 3.967	2.829	5.592	–58.9
**3a**	2.1	139.6	4.148	2.379, 3.611	2.560	4.939	–44.0
**2n**	0.042	81.6	3.861	2.399, 3.627	2.596	4.995	–34.2
**3b**	NA	148.3	4.659	2.750, 3.962	2.849	5.599	–21.1
**2o**^c^	0.020	104.1	3.134	2.760, 3.975	2.819	5.579	–67.6
**2q**^c^	18.2	89.4	4.500	3.130, 4.319	3.286	6.416	–58.4

a HNE inhibitory activity.

b The length of the proton transfer channel was calculated as *L*=*d*_3_+min(*d*_2_).

c According to the docking results, a Michaelis complex with Ser195 is formed with participation of the ester carbonyl group.

**Figure 3.**
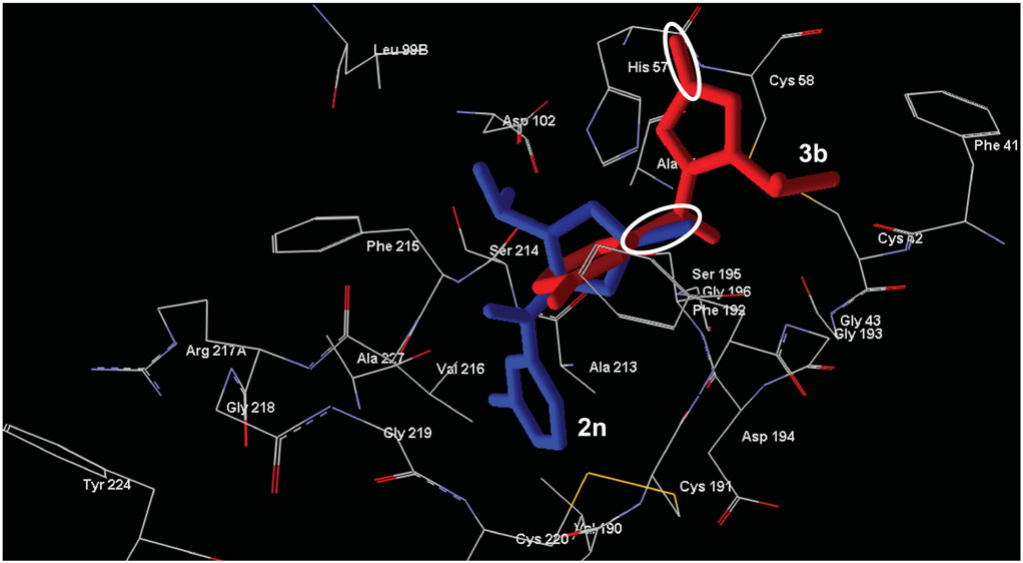
Docking poses of compounds **2n** (lower compound) and **3b** (upper compound). The ovals represent the C=S and C=O fragment of the two compounds, respectively. Residues within 6 Å from the co-crystallized ligand are shown.

Compounds **2l**, **2n**, **2o**, **2q**, **3a**, and **3b** occupy an area in the HNE binding site in the vicinity of the terminal part of the co-crystallised peptide. As presented in Table 2, the sulphur-containing compounds **3a** and **3b** had larger distances d_1_ (4.148 Å and 4.659 Å, respectively) than their counterparts **2l** and **2n** and were characterised by an angle *α* that was not suitable for easy formation of a Michaelis complex (139.6° and 148.3°, respectively). The different orientation within the binding site of sulphur compounds, with respect to compounds containing the 5-carbonyl group could also be caused by higher values of C=S bond length and radius of the sulphur atom (Figure 3).

**Figure 4.**
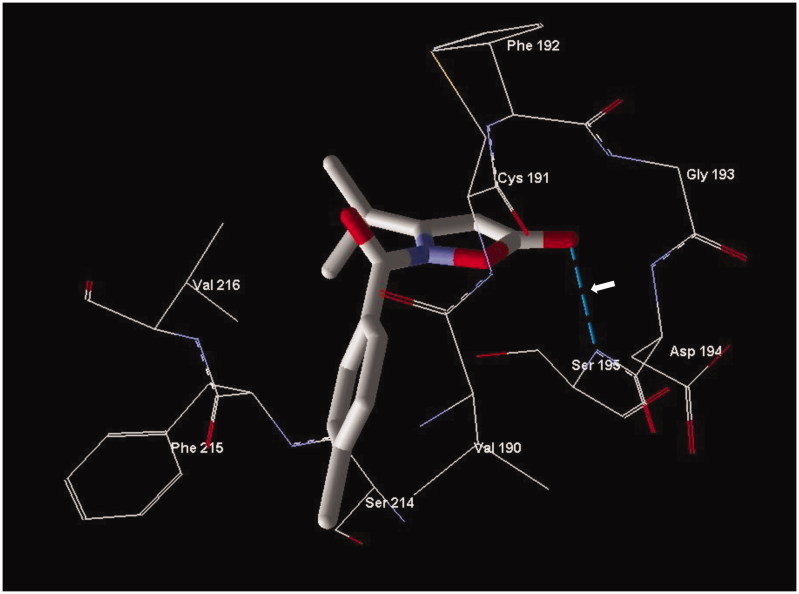
Docking pose of compound **2o**. Residues within 3 Å of the pose are visible. The H-bond is shown by a dashed line and is indicated by the arrow.

From the docking studies, it is interesting to note that these isoxazolone compounds show involvement of the endocyclic carbonyl group in formation of a Michaelis adduct, but also the amide carbonyl group is important for proper anchoring of the ligands to the enzyme pocket. For example, compounds **2o** and **2q** show values of angle *α* in the exact range (Table 2) and are located similarly in the HNE binding site to allow formation a Michaelis complex with the endocyclic carbonyl group. In particular, inhibitor **2o**, which has the best inhibitory activity (IC_50_=20 nM) forms a hydrogen bond between the oxygen of the 5-carbonyl group and the NH group of Ser195 (Figure 4) that likely improves the enzyme-ligand orientation, in addition to the involvement of the 5-carbonyl carbon atom and the Ser195 hydroxyl group, for the formation of the Michaelis complex.

In compound **2q**, the 5-carbonyl group (carbon atom) is located at a longer distance d_1_ from Ser195 than that of **2o** (see Table 2). This is due to the H-bond interaction of the oxygen atom of the 5-carbonyl group that involves the NH group of Gly193 instead of the NH group of Ser195. Moreover, for derivative **2q**, the imidazole ring of His57 is rotated unfavourably, thus enhancing the length *L* of the proton transfer channel (Table 2). Consequently, the conditions for formation of a Michaelis complex are not as suitable and lead to reduced inhibitory activity (IC_50_=18.2 μM). Differences between **2o** and **2q**, which are p-tolyl- and p-cyanophenyl derivatives, respectively, could also likely be related to variations in hydrophobicity, polarity of substituents, and different orientation of flexible side chains in the ligand–enzyme complex. Thus, our docking studies indicate that the 2-amido carbonyl group also plays an important role in positioning the ligand within the binding site, although the attack of the Ser195 is directed at the endocyclic C=O group (5-carbonyl group).

The sum of non-bonding interaction energies between HNE and the two atoms of the amido carbonyl moiety of **2o** equals –12.86 kcal/mol, which is even more negative than for the endocyclic carbonyl (–9.11 kcal/mol), indicating a strong interaction with the enzyme. Non-specific Van der Waals interaction by the tolyl ring and/or polar interactions by the 2-amido group are responsible for proper anchoring of **2o** to the sub-pocket of the binding site surrounded by Cys191, Phe192, Phe215, Val216, and Cys220.

Although **2q** shows these nonspecific interactions with the sub-pocket binding site, the different orientation in the enzyme pocket caused by the H-bond interaction of the oxygen atom of the 5-carbonyl group involving the NH Gly193 (see above) could explain the lower inhibitory activity compared to **2o**.

### Stability and kinetic features

**Figure 5.**
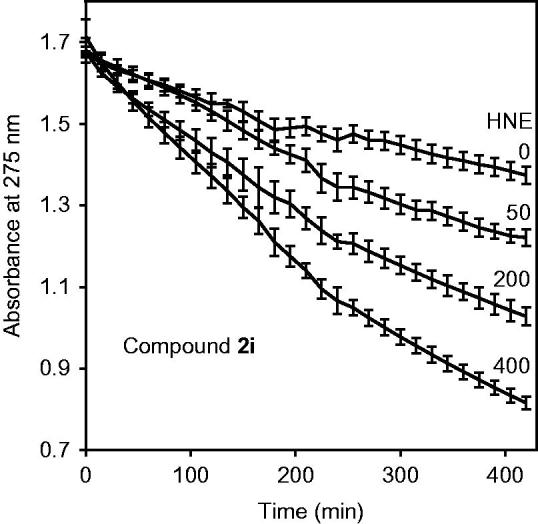
Analysis of compound **2i** spontaneous hydrolysis. Compound **2i** (80 μM) was incubated in 0.05 M phosphate buffer (pH 7.5, 25 °C) supplemented with 0, 50, 200, and 400 μg/ml HNE, as indicated. Spontaneous hydrolysis was monitored by measuring changes in absorbance at 275 nm (absorption maximum of compound **2i**) over time. The data are presented as the mean ± SD of triplicate samples from one experiment, which is representative of two independent experiments.

The most potent seven isoxazolones with IC_50_<100 nM were further evaluated for chemical stability in aqueous buffer using spectrophotometry to detect compound hydrolysis. The compounds had *t*_1/2_ values from 3.1 to 19.3 h for spontaneous hydrolysis (Table 3), indicating that these isoxazolones were more stable than our previously described HNE inhibitors with *N*-benzoylindazole scaffolds^21^^,^^22^. We also tested compound hydrolysis in the presence of HNE and found that the speed of hydrolysis gradually increased with increasing enzyme concentration. As an example, Figure 5 shows dose-dependent hydrolysis of compound **2i** over time (Figure 5). In the presence of 400 mU/ml HNE, the hydrolysis was 2.2–4.8-fold higher than for spontaneous hydrolysis (i.e. without HNE) (Table 3).

**Table 3.** Half-life (*t*_1/2_) for the hydrolysis of selected isoxazolone derivatives in aqueous buffer and in the presence of HNE.

		*t*_1/2_ (h)	
Compd.	Max (nm)^a^	Spontaneous hydrolysis	In the presence HNE^b^
**2d**	290	7.7	3.5
**2i**	275	19.3	4.0
**2j**	285	5.3	2.1
**2l**	275	19.3	4.3
**2n**	285	6.2	2.6
**2o**	290	8.9	3.4
**2s**	285	3.1	0.9

a Absorption maximum of the compounds for monitoring hydrolysis.

b Hydrolysis was monitored in the presence of 400 mU/ml HNE.

**Figure 6.**
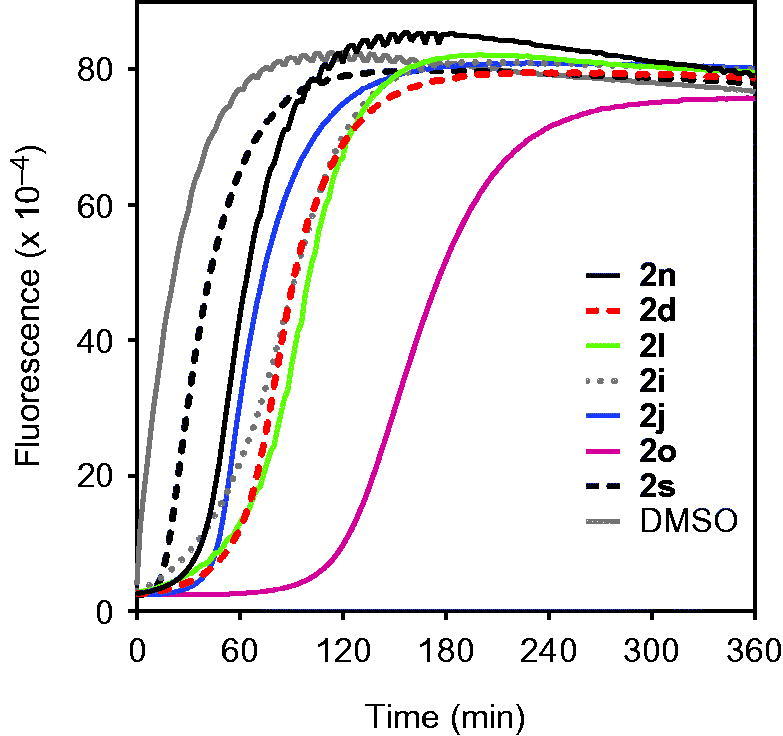
Evaluation of HNE inhibition by selected isoxazolone derivatives. HNE was incubated with the indicated compounds at 5 μM concentrations, and kinetic curves monitoring substrate cleavage catalysed by HNE are shown. Representative curves are from three independent experiments.

**Figure 7.**
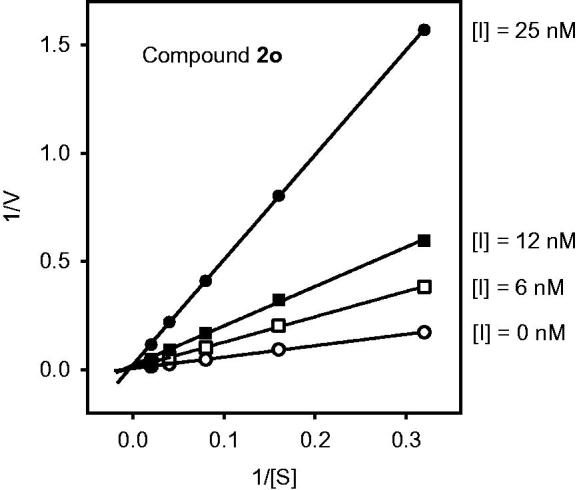
Kinetics of HNE inhibition by compound **2o**. Representative double-reciprocal Lineweaver–Burk plot from three independent experiments.

We also evaluated reversibility of the HNE-inhibitor complex over time for most potent compounds. As shown in Figure 6, HNE inhibition was maximal during the first 30 min for compounds **2n**, **2d**, **2l**, **2i**, and **2j**. However, inhibition by compound **2s** was reversed after 10 min. The most stable inhibition was found for **2o**, where inhibition of HNE activity was reversed only after 2 h (Figure 6). To better understand the mechanism of action of this isoxazolone HNE inhibitor, we performed additional kinetic experiments. As shown in Figure 7, the representative double-reciprocal Lineweaver–Burk plot of fluorogenic substrate hydrolysis by HNE in the absence and presence of compound **2o** indicates that this compound is a competitive HNE inhibitor.

## Conclusions

In conclusion, the present study demonstrates that the isoxazolone nucleus is an appropriate scaffold for the development of potent HNE inhibitors. The most potent compounds were the 4-unsubstituted derivatives. In particular, compounds **2o** and **2s**, had IC_50_ values of 20 nM and 34 nM, respectively. Docking studies suggest that, differently from our previous N-benzoylindazoles compounds^21^^,^^22^, the attack of Ser195 is directed at the endocyclic C=O group at position *5* but also that the amidic C=O group is important for anchoring to the sub-pocket of the binding site surrounded by Cys191, Phe192, Phe215, Val216, and Cys220 through nonspecific van der Waals and polar interactions. This hypothesis is in agreement with the inactivity of the *N*-alkyl/aryl derivatives and the thioanalogues. Studies of chemical stability indicated that the new isoxazolones are more stable than the compounds we previously studied^21–24^, with *t*_1/2_ values from 3.1 to 19.3 h, and that they were competitive HNE inhibitors. Thus, this new class of HNE inhibitors has great potential for the development of potent and relatively stable HNE inhibitors.

## Supplementary Material

IENZ_1326915_Supplementary_Material.pdf
