# Supplementary material for: Isoxazol-5(2H)-one: a new scaffold for potent human neutrophil elastase (HNE) inhibitors
Source: J Enzyme Inhib Med Chem. 2017 Jun 14;32(1):821–31. doi: 10.1080/14756366.2017.1326915 (PMC5927774; doi:10.1080/14756366.2017.1326915)
Supplement: IENZ_1326915_Supplementary_Material.pdf [file IENZ_A_1326915_SM4174.pdf]

SUPPLEMENTAL MATERIAL FOR

Isoxazol-5(2*H*)-one: a new scaffold for potent human neutrophil elastase (HNE) inhibitors

Claudia Vergelli[a], *Igor A. Schepetkin*[b], *Letizia Crocetti*[a*], *Antonella Iacovone*[a], *Maria Paola Giovannoni*[a], *Gabriella Guerrini*[a], *Andrei I. Khlebnikov*[c], *Samuele Ciattini*[d], *Giovanna Ciciani*[a], *Mark T. Quinn*[b]

[a]NEUROFARBA, Sezione di Farmaceutica e Nutraceutica, Università degli Studi di Firenze, Via Ugo Schiff 6, 50019 Sesto Fiorentino, Italy.

[2]Department of Microbiology and Immunology, Montana State University, Bozeman, MT 59717, USA.

[c]Department of Biotechnology and Organic Chemistry, Tomsk Polytechnic University, Tomsk 634050, Russia and Department of Chemistry, Altai State Technical University, Barnaul, Russia.

[d]Dipartimento di Chimica, Centro di Cristallografia, Università degli Studi di Firenze, Via della Lastruccia 3, 50019 Sesto Fiorentino, Italy.

Table of contents

1. ^{1}H-NMR, ^{13}C-NMR, HMBC and HSQC spectra of compounds **2a, 2d, 2e, 2n, 2s, 4a** and **4c**.
2. Crystal data and structure refinement for **2j** (**Table S1**)
3. Atomic coordinates (x 10^4) and equivalent isotropic displacement parameters (Å^2 x 10^3) for **2j** (**Table S2**)
4. Anisotropic displacement parameters (Å^2 x 10^3) for **2j** (**Table S3**)
5. Hydrogen coordinates (x 10^4) and isotropic displacement parameters (Å^2 x 10^3) for **2j** (**Table S4**)
6. Molecular formula strings (**Table S5**)
7. Elemental analysis (**Table S6**)

Compound 2a

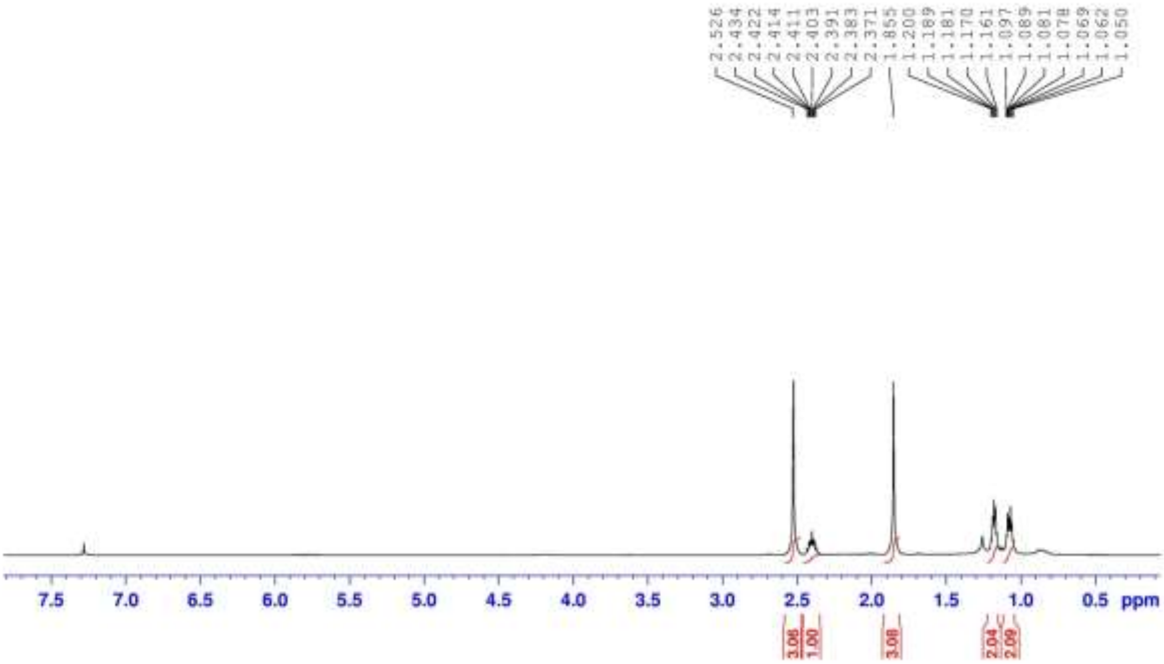

13C Comp. 2a

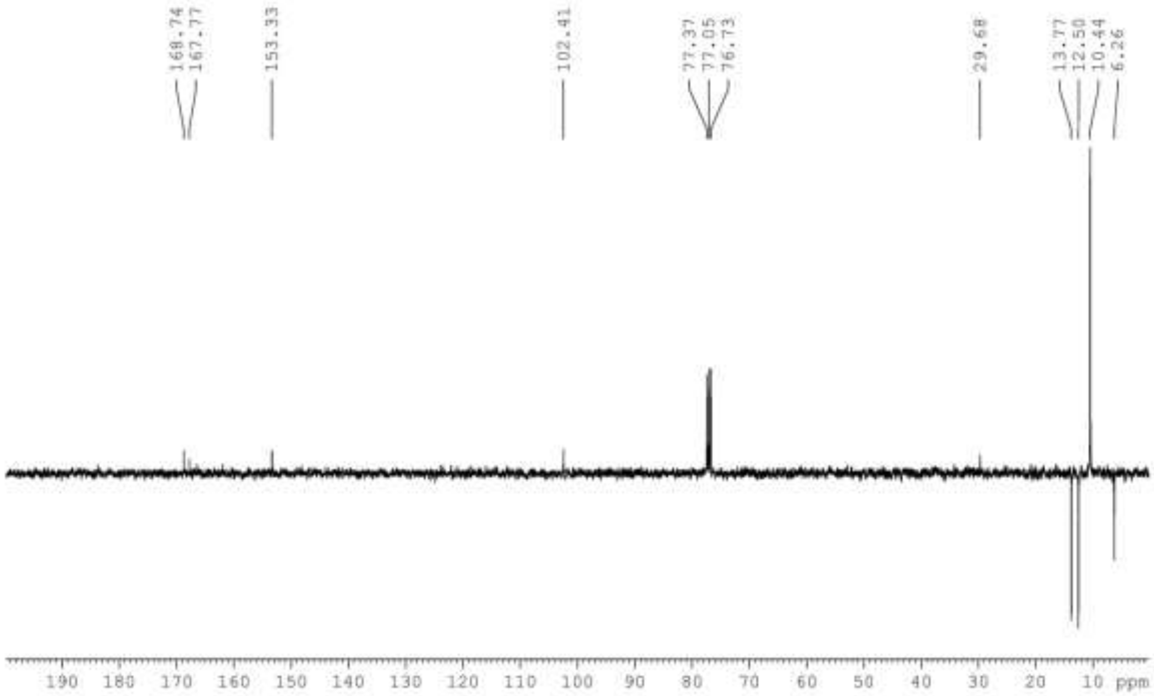

HMBC Comp. 2a

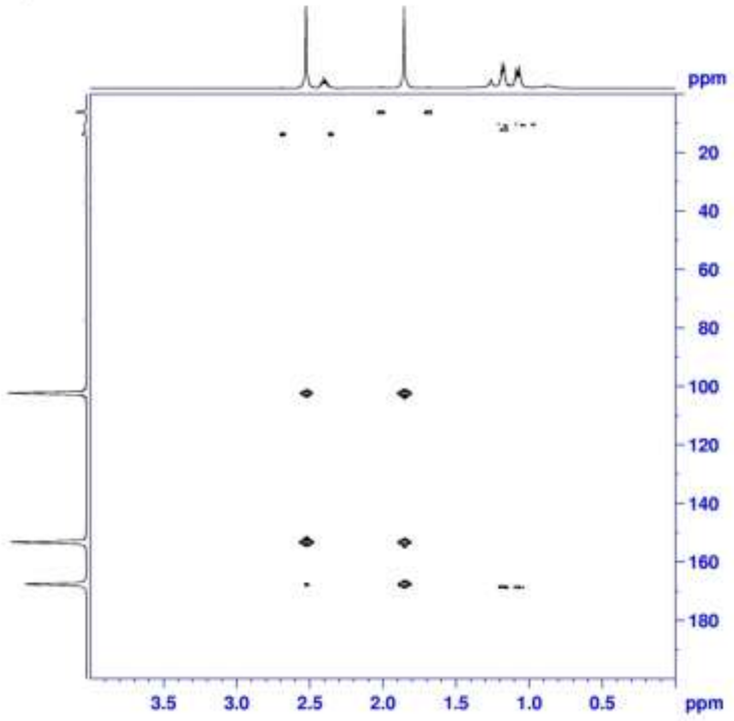

HSQC Comp. 2a

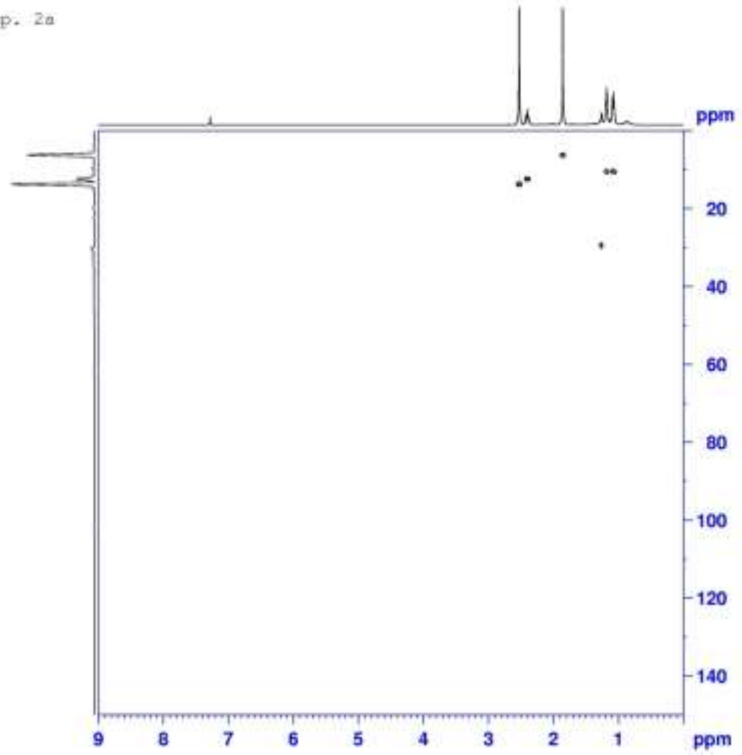

Compound 2d

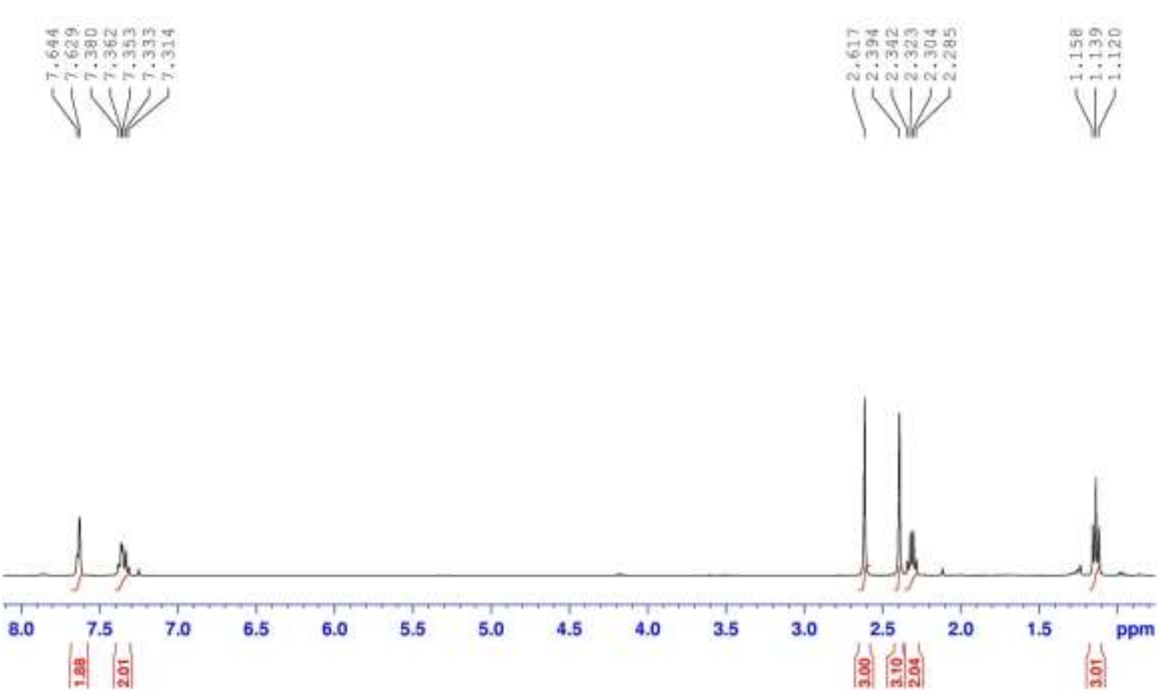

13C Comp. 2d

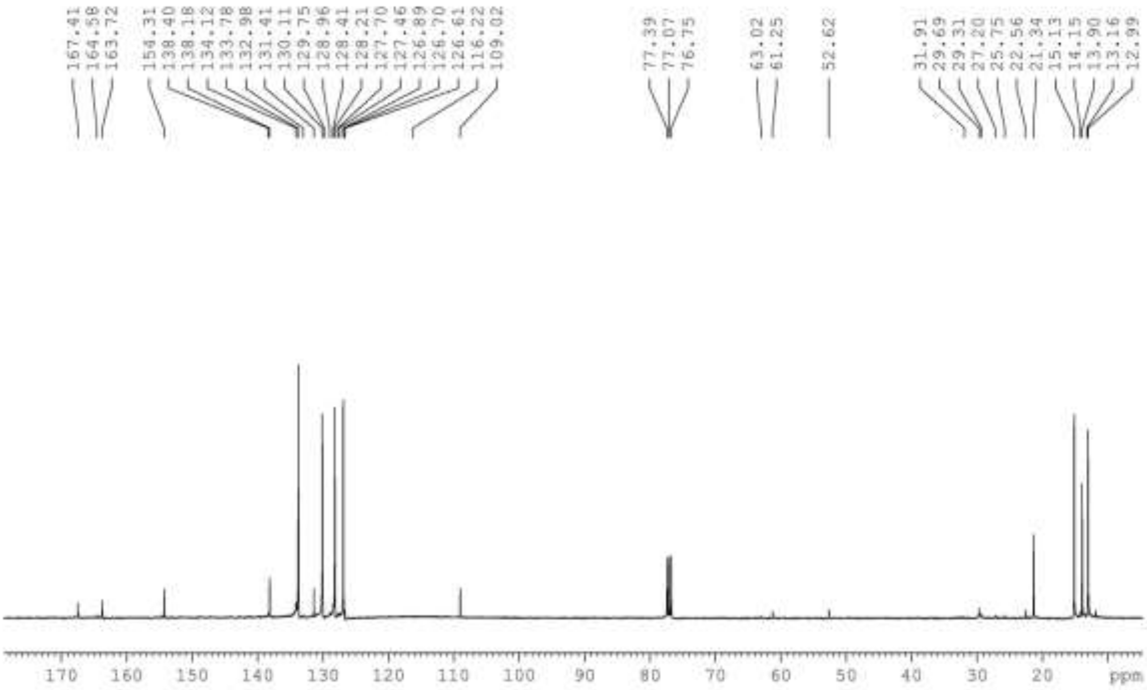

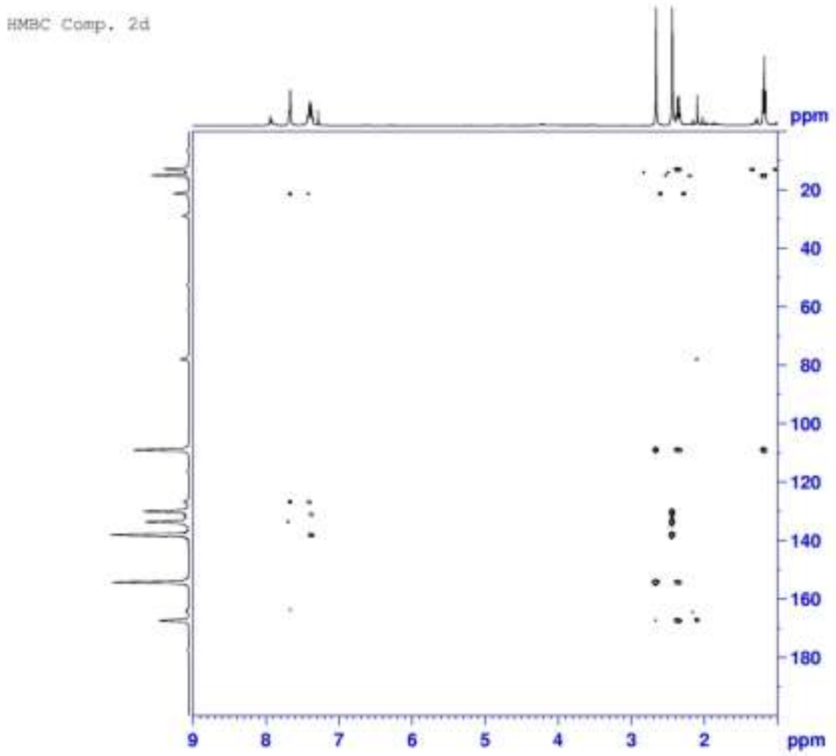
HMBC Comp. 2d
ppm
20
40
60
80
100
120
140
160
180
9
8
7
6
5
4
3
2
ppm

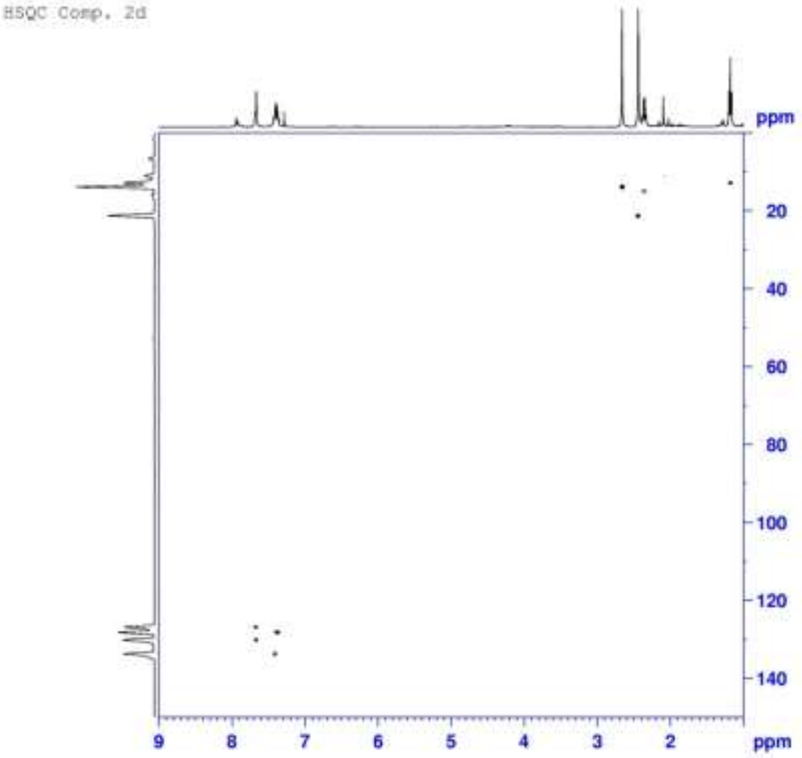
HSQC Comp. 2d
ppm
20
40
60
80
100
120
140
9
8
7
6
5
4
3
2
ppm

Compound 2e

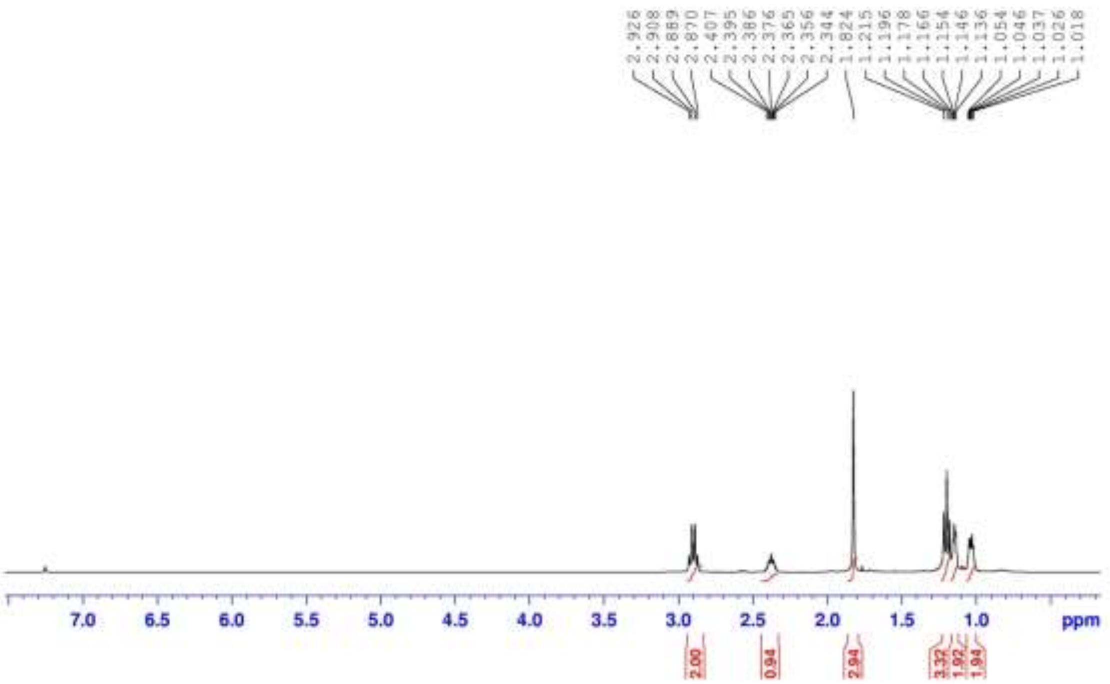

Compound 2e

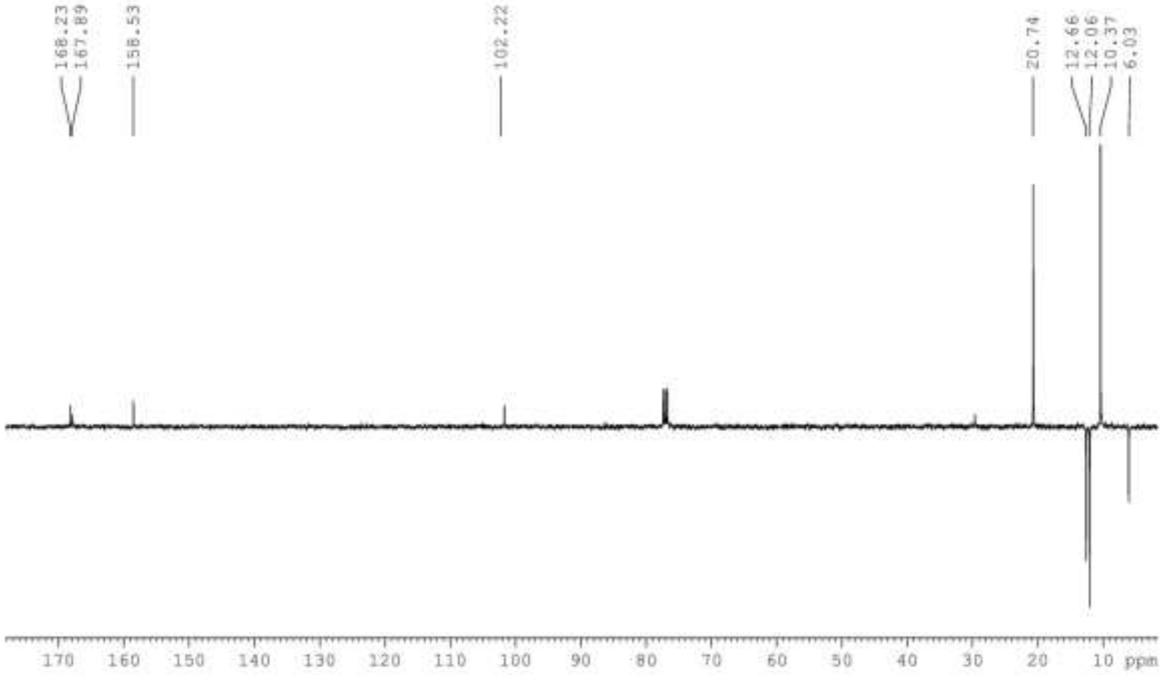

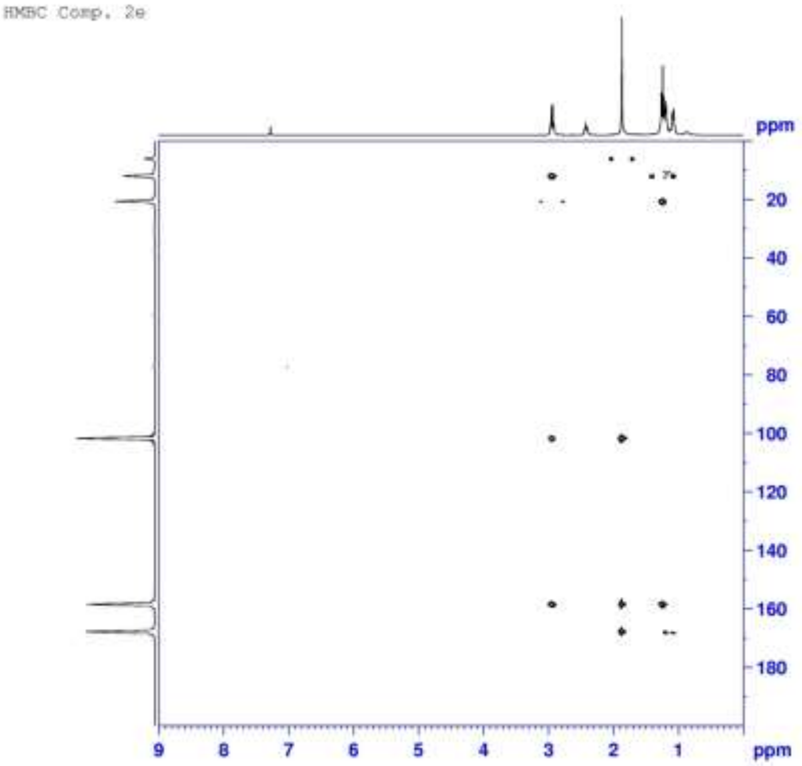
HMBC Comp. 2e
ppm
20
40
60
80
100
120
140
160
180
9
8
7
6
5
4
3
2
1
ppm

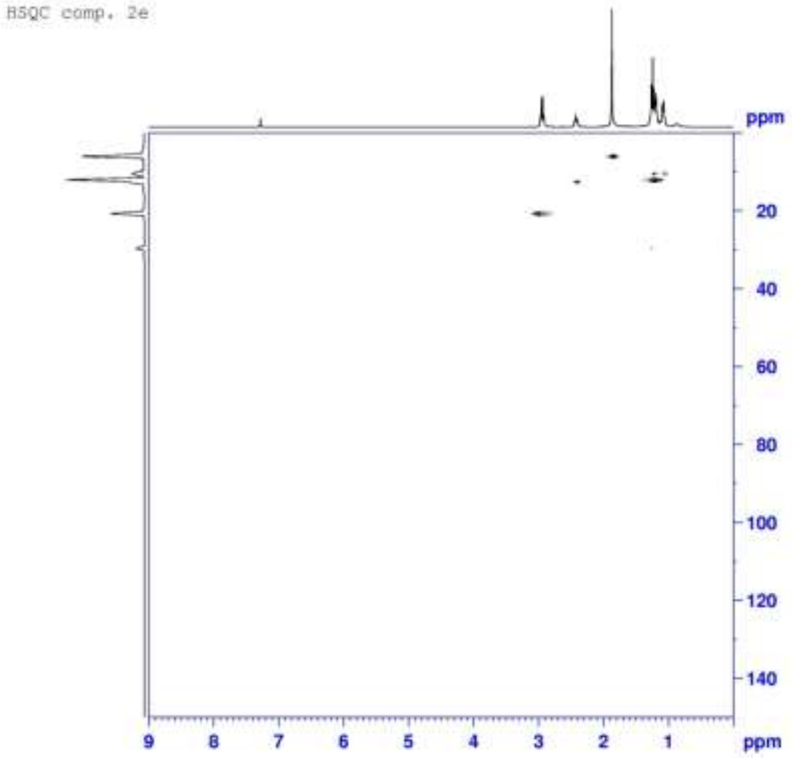
HSQC comp. 2e
ppm
20
40
60
80
100
120
140
9
8
7
6
5
4
3
2
1
ppm

Compound 2n

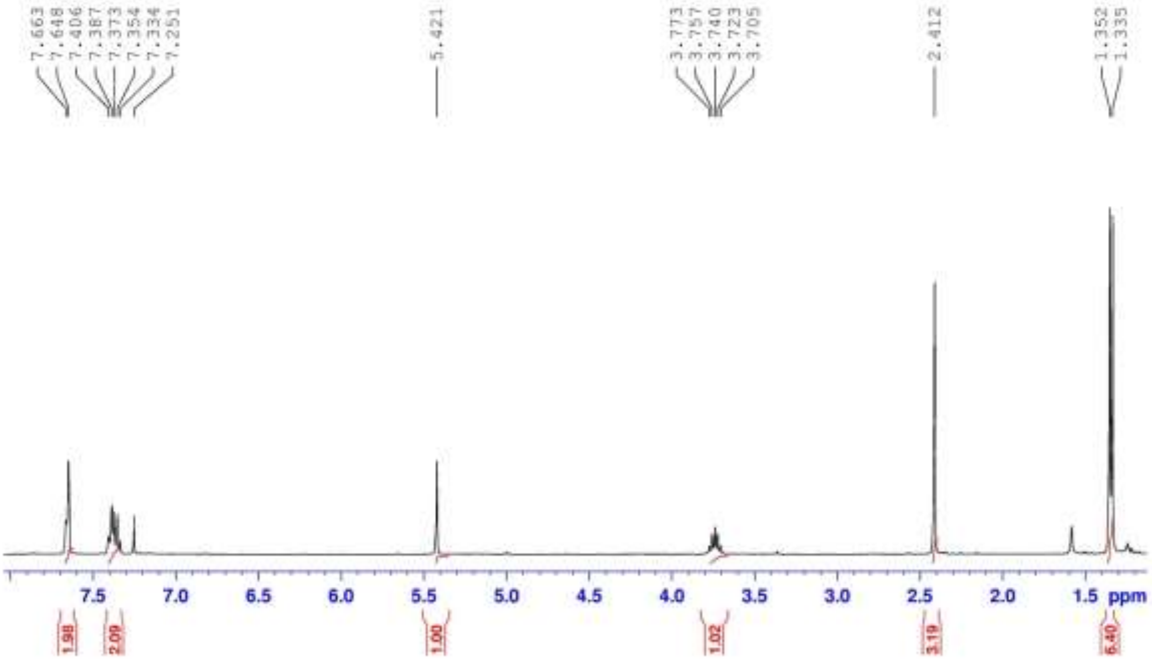

13C Comp. 2n

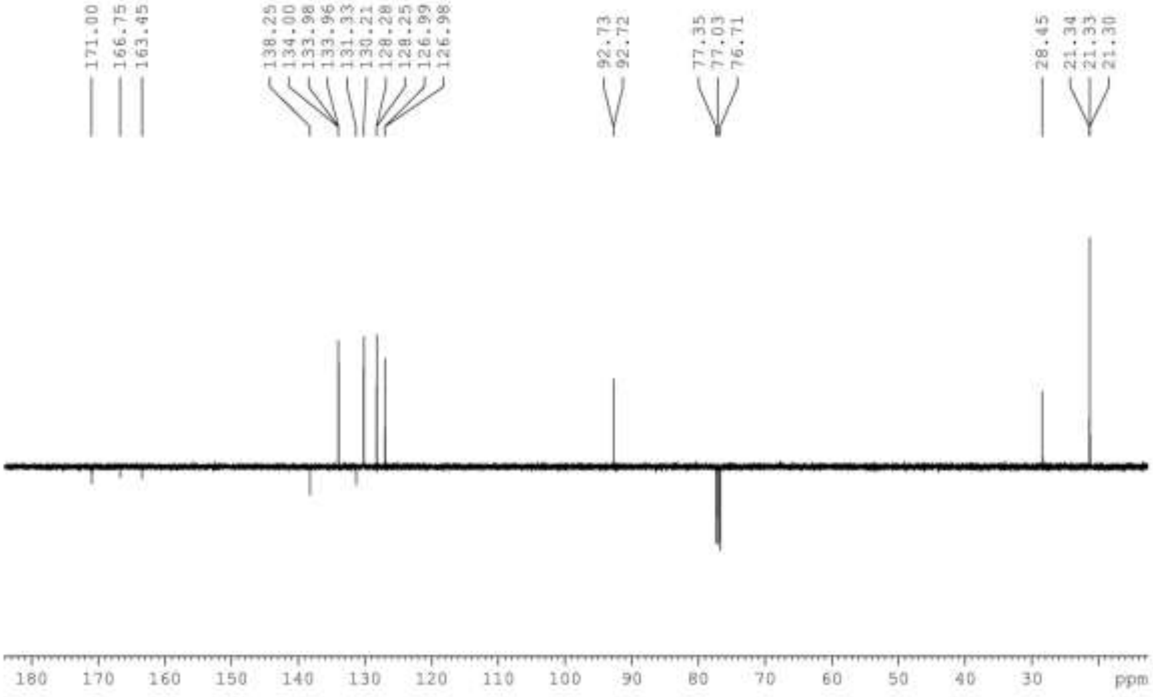

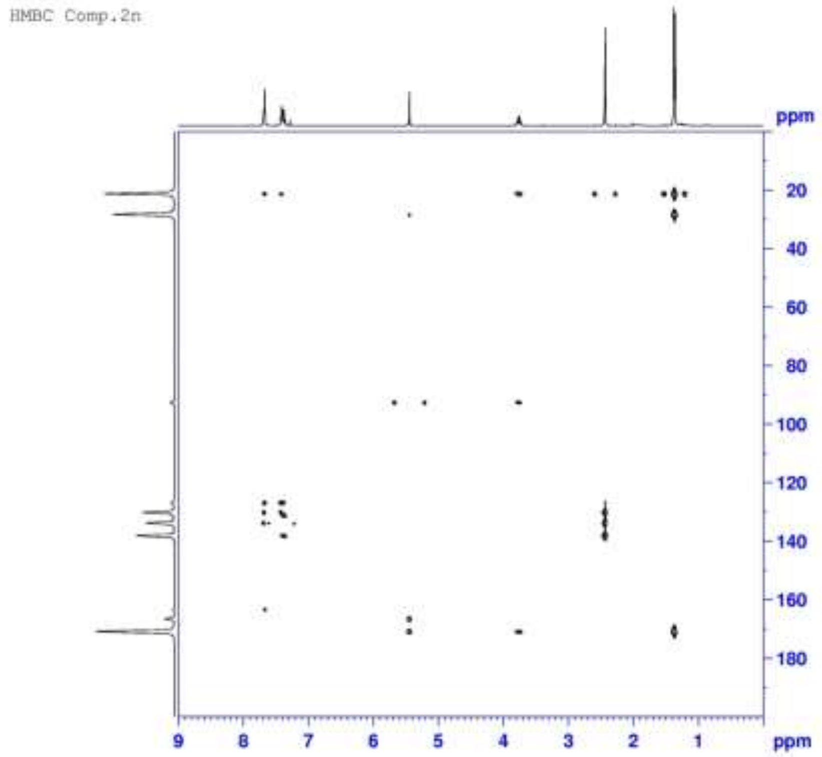
HMBC Comp.2n
ppm
20
40
60
80
100
120
140
160
180
9
8
7
6
5
4
3
2
1
ppm

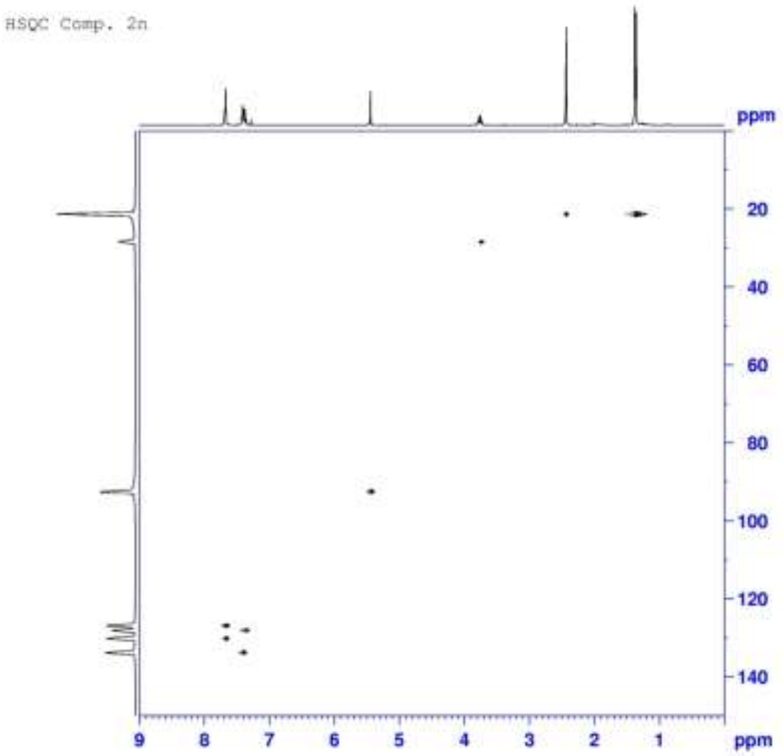
HSQC Comp. 2n
ppm
20
40
60
80
100
120
140
9
8
7
6
5
4
3
2
1
ppm

Compound 2s

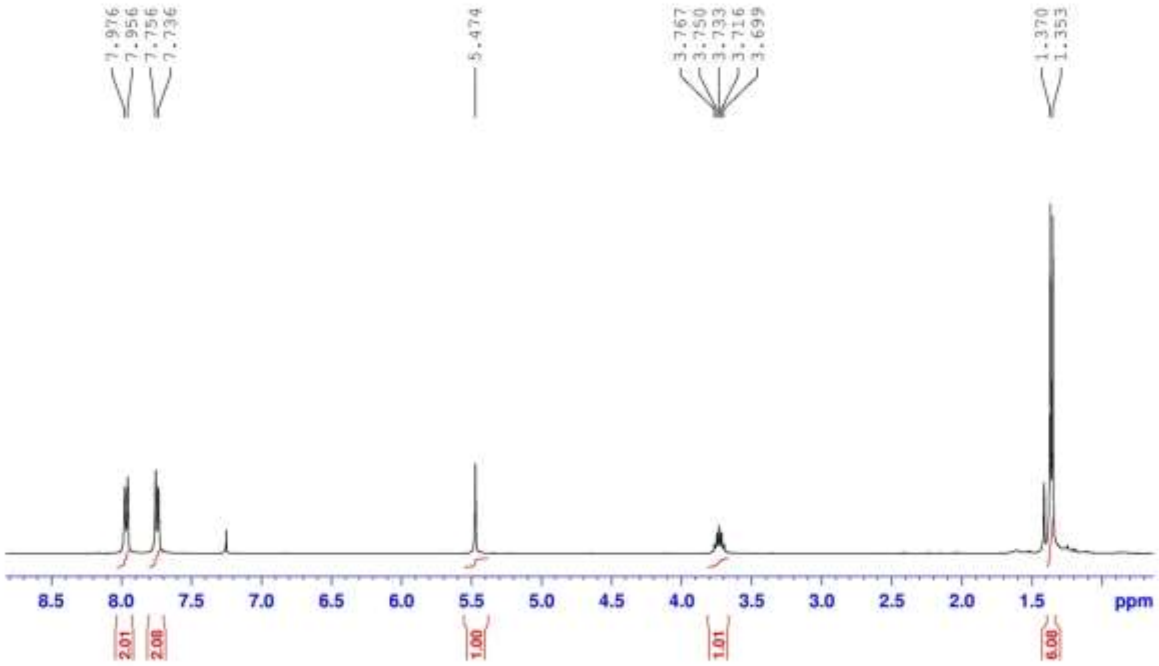

13C Comp. 2s

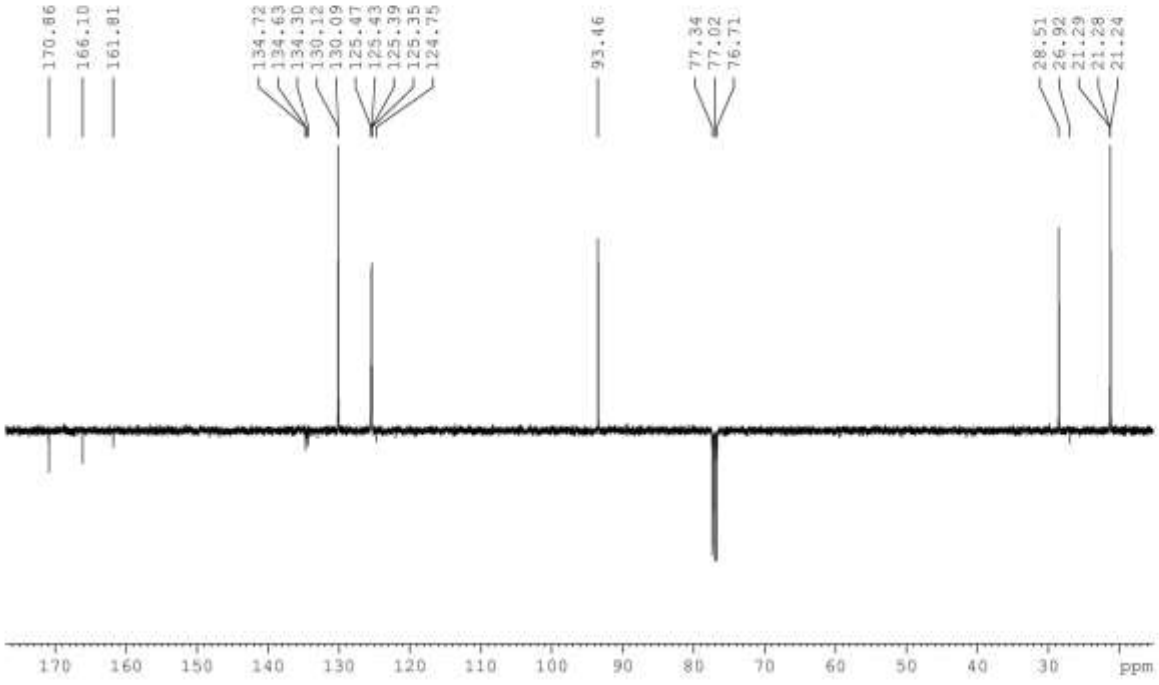

HMBC Comp. 2s

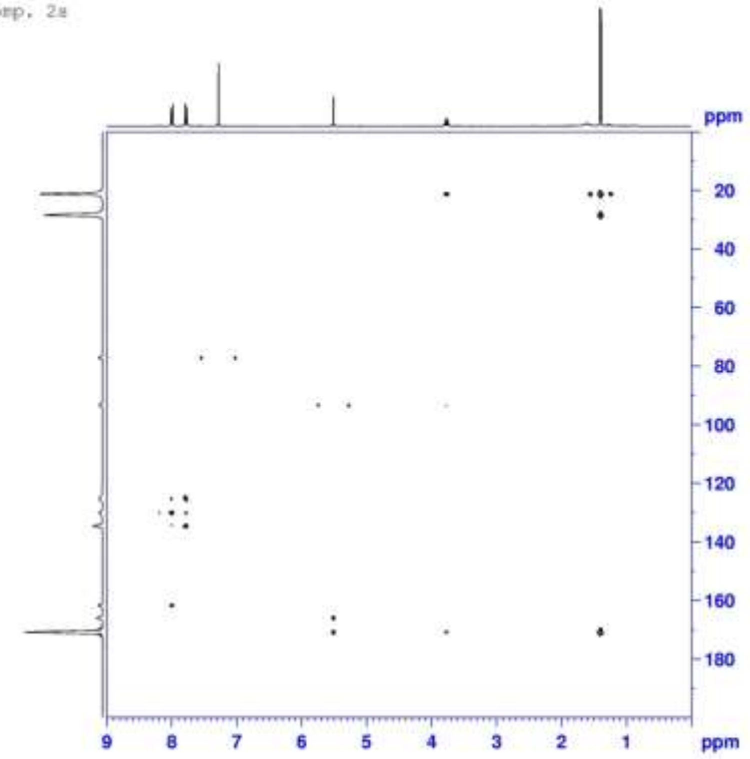

HSQC Comp. 2s

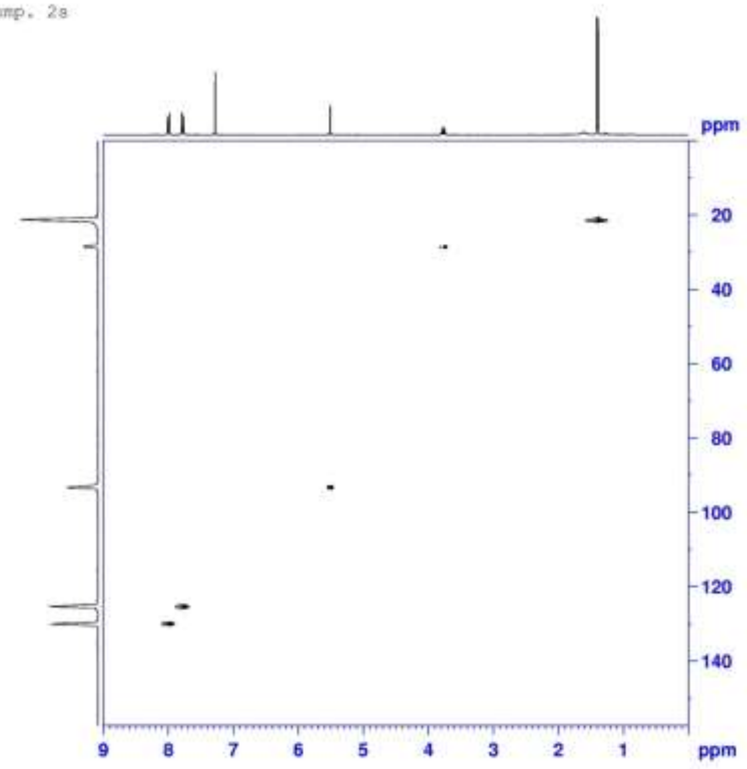

Compound 4a

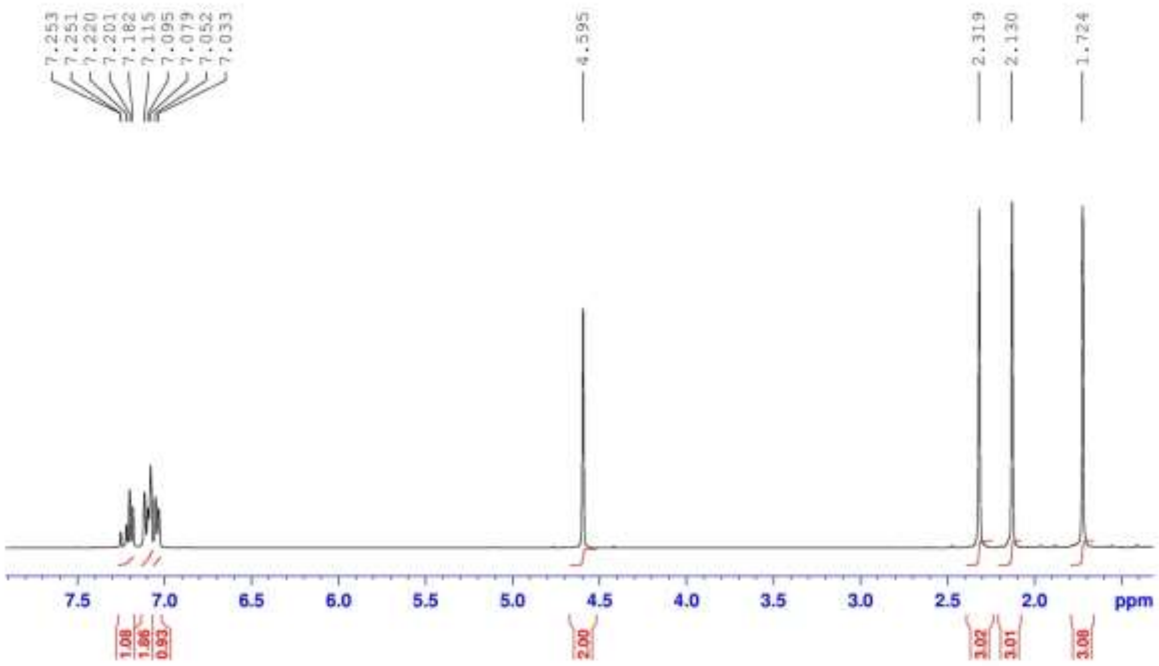

13C Comp. 4a

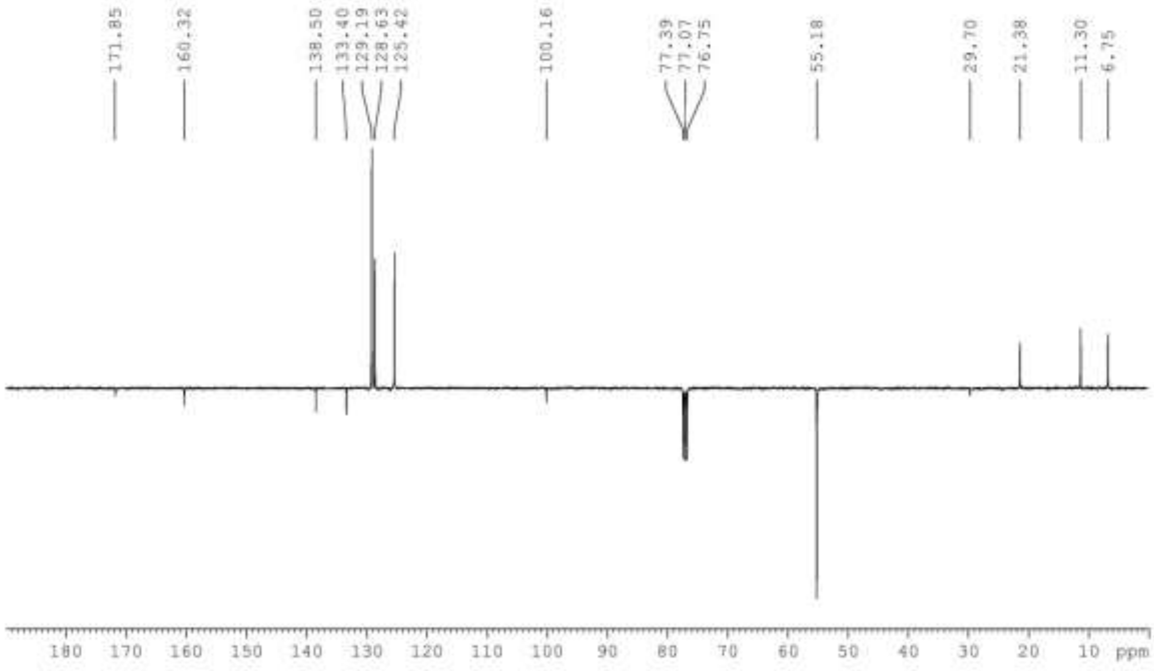

HMBC Comp. 4a

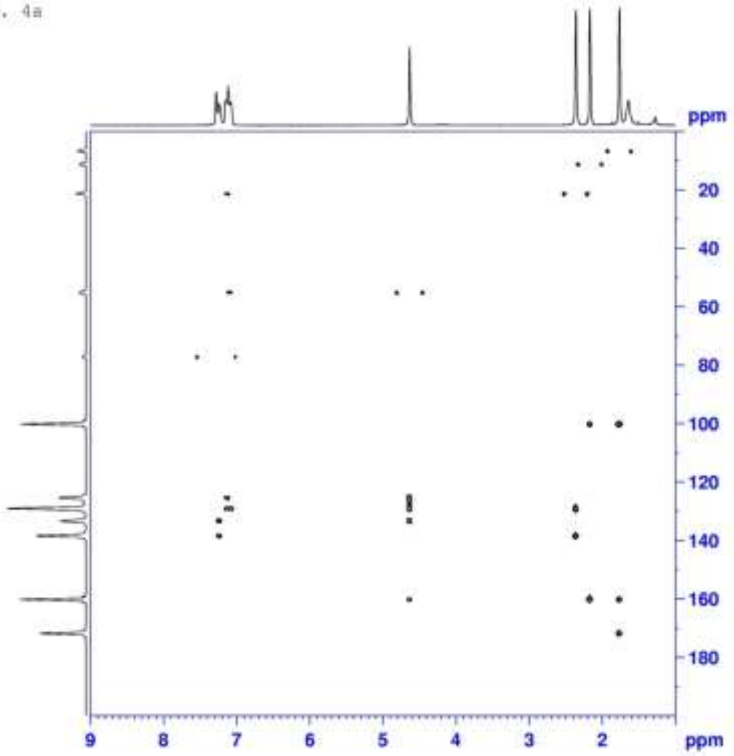

HSQC Comp. 4a

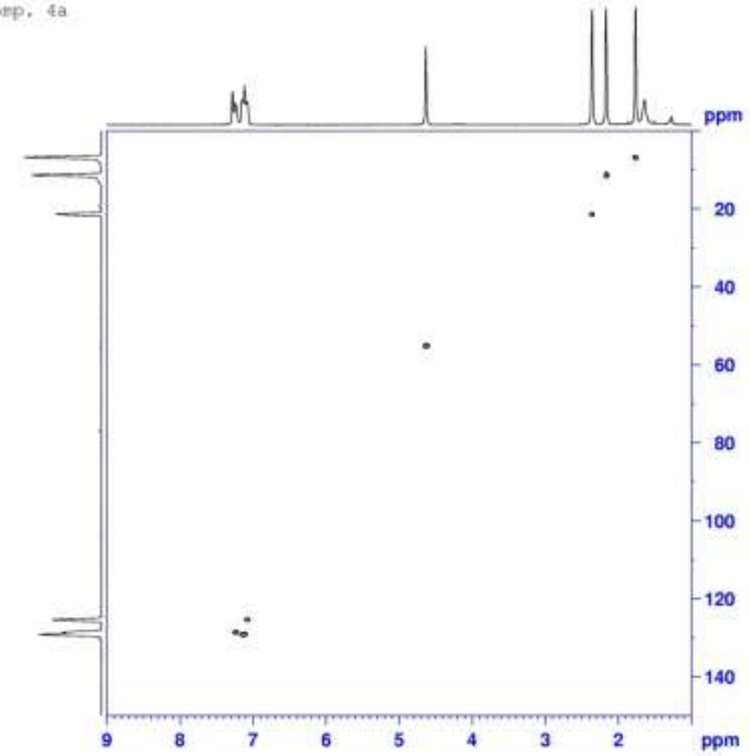

Compound 4c

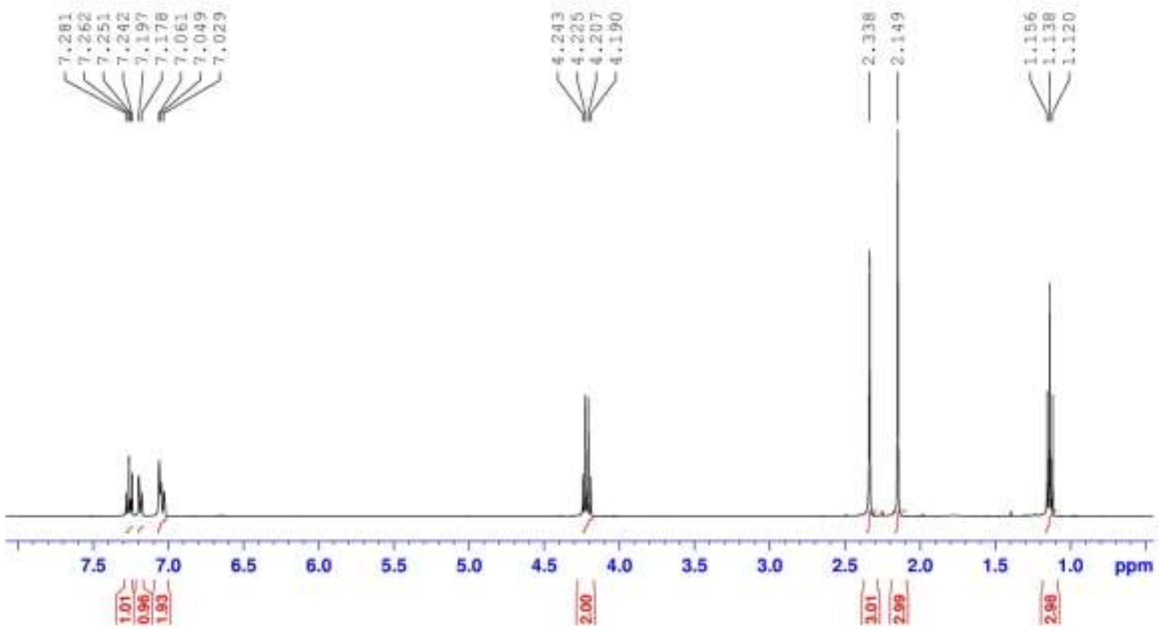

13C Comp. 4c

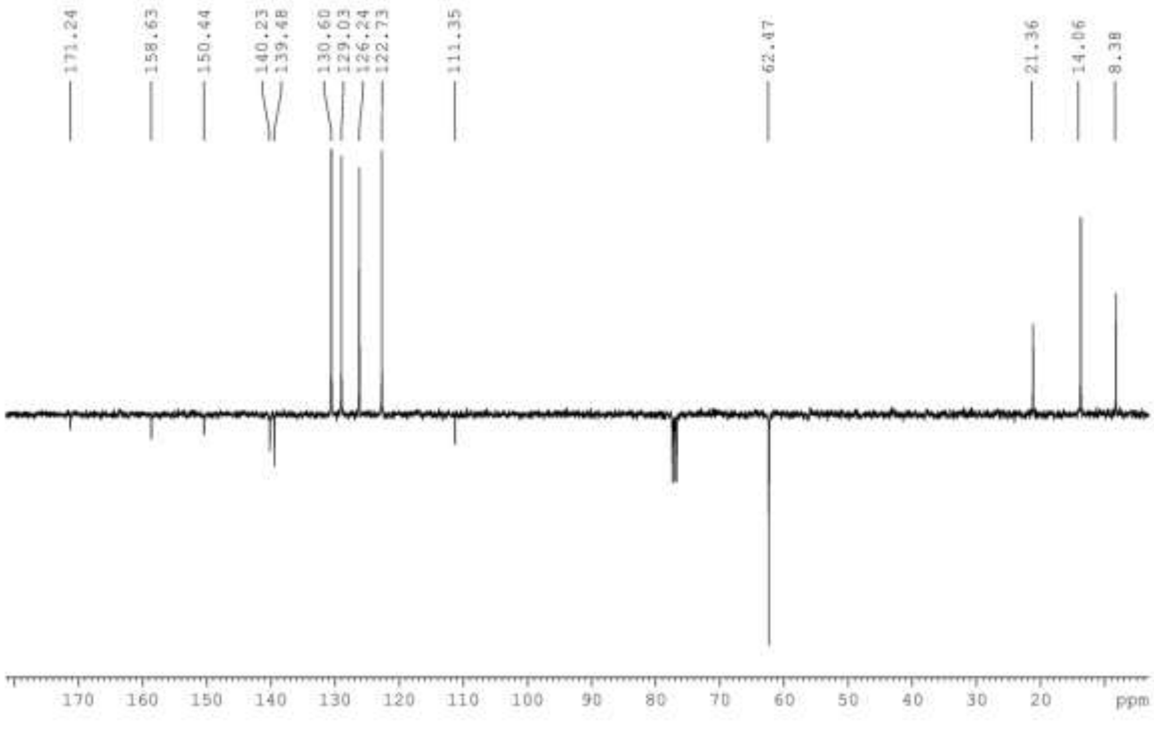

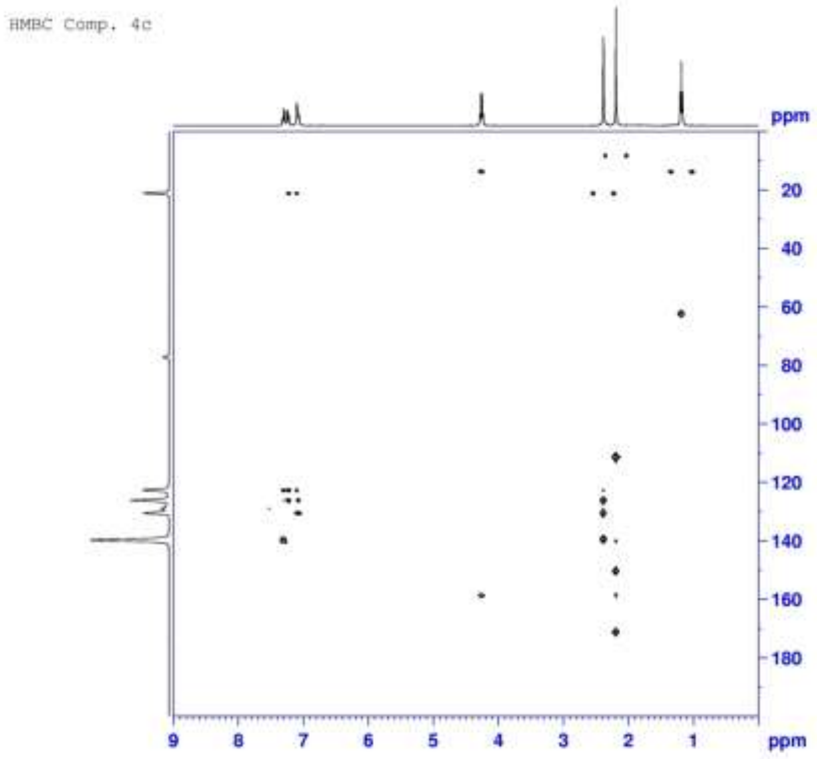
HMBC Comp. 4c
ppm
20
40
60
80
100
120
140
160
180
9
8
7
6
5
4
3
2
1
ppm

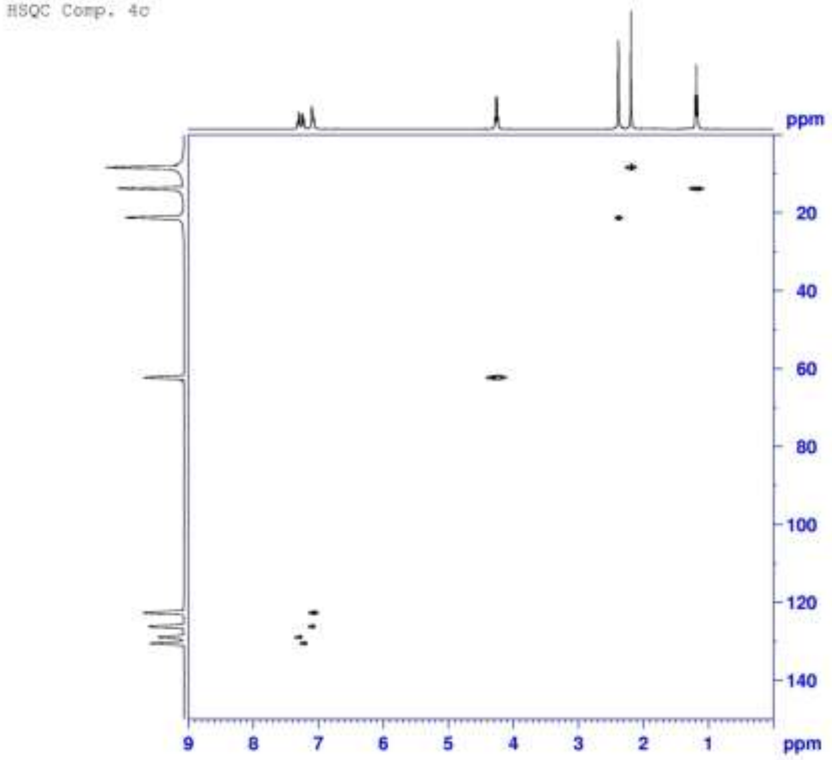
HSQC Comp. 4c
ppm
20
40
60
80
100
120
140
9
8
7
6
5
4
3
2
1
ppm

Table S1. Crystal data and structure refinement for **2j**

Empirical formula	C13 H13 N O3
Formula weight	231.24
Temperature	298(2) K
Wavelength	1.54184Å
Crystal system	Monoclinic
space group	P 21/c
Unit cell dimensions	a = 9.7480(2) Å alpha = 90°.
	b = 14.0846(3) Å beta = 91.372(2)°.
	c = 8.10170(10) Å gamma = 90°.
Volume	1112.02(4) $Å^3$
Z	4
Calculated density	1.381 Mg/m^3
Absorption coefficient	0.814 mm^{-1}
F(000)	488
Crystal size	0.35 x0.25x0.22mm
Theta range for data collection	5.520 to 72.227 deg.
Limiting indices	-11<=h<=11, -16<=k<=16, -9<=l<=7
Reflections collected / unique	6174 / 2114 [R(int) = 0.0276]
Completeness to theta =	70.000 98.9 %
Refinement method	Full-matrix least-squares on F^2
Data / restraints / parameters	2114 / 0 / 206
Goodness-of-fit on F^2	1.100
Final R indices [I>2sigma(I)]	R1 = 0.0386, wR2 = 0.0937
R indices (all data)	R1 = 0.0498, wR2 = 0.1017
Largest diff. peak and hole	0.203 and -0.349 $e.A^-$

Table S2. Atomic coordinates (x 10^4) and equivalent isotropic displacement parameters (Å^2 x 10^3) for **2j**.

U(eq) is defined as one third of the trace of the orthogonalized Uij tensor.

	x	y	z	U(eq)
O(2)	6704(1)	281(1)	6209(1)	18(1)
O(1)	5491(1)	2160(1)	3730(1)	23(1)
O(3)	6653(1)	-709(1)	8385(1)	24(1)
N(1)	5853(1)	1020(1)	5617(2)	17(1)
C(11)	4713(2)	1092(1)	6603(2)	16(1)
C(7)	7569(2)	1417(1)	1733(2)	17(1)
C(6)	7577(2)	1316(1)	3453(2)	17(1)
C(8)	6254(2)	1539(1)	4246(2)	17(1)
C(10)	4841(2)	449(1)	7829(2)	18(1)
C(2)	8764(2)	1304(1)	852(2)	18(1)
C(1)	8748(2)	1380(1)	-1000(2)	21(1)
C(5)	8800(2)	1117(1)	4310(2)	19(1)
C(4)	10004(2)	1029(1)	3435(2)	20(1)
C(3)	9987(2)	1117(1)	1732(2)	20(1)
C(12)	3570(2)	1767(1)	6219(2)	19(1)
C(9)	6082(2)	-75(1)	7639(2)	18(1)
C(13)	2370(2)	1628(1)	7372(2)	25(1)

Table S3. Anisotropic displacement parameters ($Å^2 \times 10^3$) for **2j**.

The anisotropic displacement factor exponent takes the form:

$-2 pi^2 [h^2 a^{*2} U11 + ... + 2 h k a^* b^* U12]$

	U11	U22	U33	U23	U13	U12
O(2)	19(1)	17(1)	18(1)	4(1)	2(1)	3(1)
O(1)	23(1)	24(1)	22(1)	6(1)	2(1)	6(1)
O(3)	23(1)	25(1)	24(1)	9(1)	2(1)	2(1)
N(1)	17(1)	16(1)	17(1)	3(1)	2(1)	4(1)
C(11)	17(1)	17(1)	16(1)	-6(1)	1(1)	-3(1)
C(7)	18(1)	13(1)	19(1)	1(1)	0(1)	-2(1)
C(6)	18(1)	13(1)	19(1)	0(1)	2(1)	-2(1)
C(8)	20(1)	16(1)	14(1)	-1(1)	-1(1)	-1(1)
C(10)	18(1)	20(1)	17(1)	0(1)	1(1)	-2(1)
C(2)	22(1)	13(1)	18(1)	-1(1)	3(1)	-2(1)
C(1)	27(1)	19(1)	18(1)	-1(1)	5(1)	-1(1)
C(5)	21(1)	17(1)	19(1)	2(1)	-1(1)	-1(1)
C(4)	16(1)	20(1)	25(1)	2(1)	-2(1)	-1(1)
C(3)	19(1)	17(1)	24(1)	0(1)	5(1)	0(1)
C(12)	19(1)	18(1)	20(1)	-2(1)	1(1)	2(1)
C(9)	20(1)	19(1)	15(1)	0(1)	0(1)	-3(1)
C(13)	21(1)	26(1)	29(1)	1(1)	6(1)	6(1)

Table S4. Hydrogen coordinates (x 10^4) and isotropic displacement parameters ($Å^2$ x 10^3) for **2j**

	x	y	z	U(eq)
H(13A)	2660(19)	1727(13)	8570(20)	31(5)
H(12A)	3923(17)	2399(12)	6310(20)	18(4)
H(5)	8815(17)	1068(12)	5520(20)	23(4)
H(13B)	1972(18)	972(13)	7280(20)	23(4)
H(12B)	3267(17)	1659(12)	5030(20)	23(4)
H(7)	6710(18)	1564(12)	1150(20)	21(4)
H(3)	10836(19)	1044(13)	1140(20)	30(5)
H(10)	4214(19)	365(13)	8720(20)	27(5)
H(4)	10859(19)	917(13)	4040(20)	27(5)
H(13C)	1655(19)	2086(13)	7100(20)	28(5)
H(1B)	9430(20)	1858(15)	-1360(30)	40(6)
H(1C)	7790(20)	1580(13)	-1440(20)	34(5)
H(1A)	8980(20)	770(15)	-1510(20)	36(5)

Table S5. Molecular formula strings

compd_ID	SMILES
**2a**	CC(N(C(C1CC1)=O)O2)=C(C)C2=O
**2b**	CC(N(C(C1=CC=CC(C)=C1)=O)O2)=C(C)C2=O
**2c**	CC1=C(CC)N(C(C2CC2)=O)OC1=O
**2d**	CC(N(C(C1=CC=CC(C)=C1)=O)O2)=C(CC)C2=O
**2e**	O=C1ON(C(C2CC2)=O)C(CC)=C1
**2f**	CC1=C(CC)N(C(C2=CC=CC(C)=C2)=O)OC1=O
**2g**	O=C1ON(C(C2CC2)=O)C(C(OCC)=O)=C1C
**2h**	O=C1ON(C(C2=CC=CC(C)=C2)=O)C(C(OCC)=O)=C1C
**3a**	S=C1ON(C(C2CC2)=O)C(C(C)C)=C1
**3b**	S=C1ON(C(C2=CC=CC(C)=C2)=O)C(C(C)C)=C1
**2i**	O=C1ON(C(C2CC2)=O)C(CC)=C1
**2j**	O=C1ON(C(C2=CC=CC(C)=C2)=O)C(CC)=C1
**2k**	O=C1ON(C(CC)=O)C(C(C)C)=C1
**2l**	O=C1ON(C(C2CC2)=O)C(C(C)C)=C1
**2m**	O=C1ON(C(C2CCCC2)=O)C(C(C)C)=C1
**2n**	O=C1ON(C(C2=CC=CC(C)=C2)=O)C(C(C)C)=C1
**2o**	O=C1ON(C(C2=CC=C(C)C=C2)=O)C(C(C)C)=C1
**2p**	O=C1ON(C(C2=CC=CC(C#N)=C2)=O)C(C(C)C)=C1
**2q**	O=C1ON(C(C2=CC=C(C#N)C=C2)=O)C(C(C)C)=C1
**2r**	O=C1ON(C(C2=CC=CC(C(F)(F)F)=C2)=O)C(C(C)C)=C1
**2s**	O=C1ON(C(C2=CC=C(C(F)(F)F)C=C2)=O)C(C(C)C)=C1
**4a**	CC(N(CC1=CC=CC=C1)O2)=C(C)C2=O
**4b**	O=C1ON(CC2=CC=CC(C)=C2)C(C(C)C)=C1
**4c**	CC1=C(C(OCC)=O)N(C2=CC=CC(C)=C2)OC1=O

Table S6. Elemental analysis

Comp.	Formula (MW)	Anal. Calcd.				Anal. Found		
		C	H	N		C	H	N
**2a**	$C_9H_{11}NO_3$ (181.19)	59.66	6.12	7.33		59.89	6.14	7.76
**2b**	$C_{13}H_{13}NO_3$ (231.25)	67.52	5.67	6.06		67.79	5.69	6.08
**2c**	$C_{10}H_{13}NO_3$ (195.22)	61.53	6.71	7.18		61.77	6.73	7.20
**2d**	$C_{14}H_{15}NO_3$ (245.27)	68.56	6.16	5.71		68.83	6.18	5.73
**2e**	$C_{10}H_{13}NO_3$ (195.22)	61.53	6.71	7.18		61.77	6.73	7.20
**2f**	$C_{14}H_{15}NO_3$ (245.27)	68.56	6.16	5.71		68.83	6.18	5.73
**2g**	$C_{11}H_{13}NO_5$ (239.22)	55.23	5.48	5.86		55.45	5.50	5.88
**2h**	$C_{15}H_{15}NO_5$ (289.28)	62.28	5.23	4.84		62.52	5.25	4.85
**2i**	$C_9H_{11}NO_3$ (181.19)	59.66	6.12	7.33		59.89	6.14	7.76
**2j**	$C_{13}H_{13}NO_3$ (231.25)	67.52	5.67	6.06		67.79	5.69	6.08
**2k**	$C_9H_{13}NO_3$ (183.20)	59.00	7.15	7.65		59.23	7.17	7.68
**2l**	$C_{10}H_{13}NO_3$ (195.22)	61.53	6.71	7.18		61.77	6.73	7.20
**2m**	$C_{12}H_{17}NO_3$ (223.27)	64.55	7.67	6.27		64.80	7.70	6.29
**2n**	$C_{14}H_{15}NO_3$ (245.27)	68.56	6.16	5.71		68.83	6.18	5.73
**2o**	$C_{14}H_{15}NO_3$ (245.27)	68.56	6.16	5.71		68.83	6.18	5.73
**2p**	$C_{14}H_{12}N_2O_3$ (256.26)	65.62	4.72	10.93		65.88	4.73	10.97
**2q**	$C_{14}H_{12}N_2O_3$ (256.26)	65.62	4.72	10.93		65.88	4.73	10.97
**2r**	$C_{14}H_{12}F_3NO_3$ (299.25)	56.19	4.04	4.68		56.41	4.05	4.69
**2s**	$C_{14}H_{12}F_3NO_3$ (299.25)	56.19	4.04	4.68		56.41	4.05	4.69

**3a**	$C_{10}H_{13}NO_2S$ (211.28)	56.85	6.20	6.63		57.07	6.22	6.65
**3b**	$C_{14}H_{15}NO_2S$ (261.34)	64.34	5.79	5.36		64.59	5.81	5.38
**4a**	$C_{13}H_{15}NO_2$ (217.26)	71.87	6.96	6.45		72.15	6.98	6.47
**4b**	$C_{14}H_{17}NO_2$ (231.29)	72.70	7.41	6.06		72.99	7.43	6.08
**4c**	$C_{14}H_{15}NO_4$ (261.27)	64.36	5.79	5.36		64.61	5.81	5.38
